# The inferior temporal cortex is a potential cortical precursor of orthographic processing in untrained monkeys

**DOI:** 10.1038/s41467-020-17714-3

**Published:** 2020-08-04

**Authors:** Rishi Rajalingham, Kohitij Kar, Sachi Sanghavi, Stanislas Dehaene, James J. DiCarlo

**Affiliations:** 1 Department of Brain and Cognitive Sciences, Massachusetts Institute of Technology, Cambridge, MA 02139 USA; 2 McGovern Institute for Brain Research, Massachusetts Institute of Technology, Cambridge, MA 02139 USA; 3 Center for Brains, Minds and Machines, Massachusetts Institute of Technology, Cambridge, MA 02139 USA; 4 Collège de France, 11 Place Marcelin Berthelot, 75005 Paris, France; 5 Cognitive Neuroimaging Unit, CEA, INSERM, Université Paris-Sud, Université Paris-Saclay, NeuroSpin Center, 91191 Gif/Yvette, France

**Keywords:** Reading, Object vision

## Abstract

The ability to recognize written letter strings is foundational to human reading, but the underlying neuronal mechanisms remain largely unknown. Recent behavioral research in baboons suggests that non-human primates may provide an opportunity to investigate this question. We recorded the activity of hundreds of neurons in V4 and the inferior temporal cortex (IT) while naïve macaque monkeys passively viewed images of letters, English words and non-word strings, and tested the capacity of those neuronal representations to support a battery of orthographic processing tasks. We found that simple linear read-outs of IT (but not V4) population responses achieved high performance on all tested tasks, even matching the performance and error patterns of baboons on word classification. These results show that the IT cortex of untrained primates can serve as a precursor of orthographic processing, suggesting that the acquisition of reading in humans relies on the recycling of a brain network evolved for other visual functions.

## Abstract (web summary)

The neuronal mechanisms underlying recognition of written letters remain unknown. Here, the authors show that populations of neurons in the ventral visual pathway of macaque monkeys encode orthographic stimuli, indicating that this pathway might be a precursor of orthographic processing abilities.

## Introduction

Literate human adults can efficiently recognize written letters and their combinations over a broad range of fonts, scripts, and sizes^1–3^. This domain of visual recognition, known as orthographic processing, is foundational to human reading abilities, because the invariant recognition of the visual word form is an indispensable step prior to accessing the sounds (phonology) and meanings (semantics) of written words^4^. It is largely unknown how orthographic processing is supported by neural populations in the human brain. Given the recency of reading and writing to the human species (a cultural invention dating to within a few thousand years), it is widely believed that the human brain could not have evolved de novo neural mechanisms for the visual processing of orthographic stimuli, and that the neural representations that underlie orthographic processing abilities must build upon, and thus be strongly constrained by, the prior evolution of the primate brain^5,6^. In particular, a dominant theory is that the ventral visual pathway, a hierarchy of cortical regions known to support visual object recognition behaviors, could be inherited from recent evolutionary ancestors and minimally repurposed (or “recycled”) through developmental experience to support orthographic processing^6^. Consistent with this hypothesis, functional imaging studies suggest that the postnatal acquisition of reading is accompanied by a partial specialization of dedicated cortical sub-regions in the human ventral visual pathway, which ultimately become strongly selective to orthographic stimuli^7–9^. However, given the limitations of human imaging methods, it has been challenging to quantitatively test if and how neural representations in the ventral visual pathway might be reused to support orthographic processing.

Interestingly, the ventral visual processing stream—a hierarchically-connected set of neocortical areas^10^—appears remarkably well conserved across many primate species, including Old-World monkeys, such as a rhesus macaques (Macaca mulatta) and baboons (Papio papio), that diverged from humans about 25 million years ago^11^. Indeed, decades of research have inferred strong anatomical and functional homologies of the ventral visual hierarchy between humans and macaque monkeys^12–14^. Previously, we observed striking similarities in invariant visual object recognition behavior between these two primate species^15,16^. Recent work suggests that non-human primates may also mimic some aspects of human orthographic processing behavior^17,18^. In particular, Grainger and colleagues showed that baboons can learn to accurately discriminate visually-presented four-letter English words from pseudoword strings^17^. Crucially, baboons were not simply memorizing every stimulus, but instead had learned to discriminate between these two categories of visual stimuli based on the general statistical properties of English spelling, as they generalized to novel stimuli with above-chance performance. Furthermore, the baboons’ patterns of behavioral performance across non-word stimuli was similar to the corresponding pattern in literate human adults, who make infrequent but systematic errors on this task. Taken together, those prior results suggest that non-human primate models, while not capturing the entirety of human reading abilities, may provide a unique opportunity to investigate the neuronal mechanisms underlying orthographic processing.

In light of this opportunity, we investigated the existence of potential neural precursors of orthographic processing in the ventral visual pathway of untrained macaque monkeys. Prior neurophysiological and neuropsychological research in macaque monkeys point to a central role of the ventral visual stream in invariant object recognition^19–21^, with neurons in inferior temporal (IT) cortex, the topmost stage of the ventral stream hierarchy, exhibiting selectivity for complex visual features and remarkable tolerance to changes in viewing conditions (e.g. position, scale, and pose)^19,22,23^. It has been suggested that such neural features could have been coopted and selected by human writing systems throughout the world^5,6,24^. Here, we reasoned that if orthographic processing abilities are supported by “recycling” primate IT cortex—either by minimal adaptations to the IT representation and/or evolutionary addition of new cortical tissue downstream of IT—then this predicts that the initial state of the IT representation, as measured in naïve macaque monkeys, should readily serve as a computational precursor of orthographic processing tests. Investigating the representation of letters and letter strings in macaque IT cortex would not only directly test this prediction but could also provide initial insights into the representation of letters and letter strings prior to reading acquisition.

**Fig. 1:**
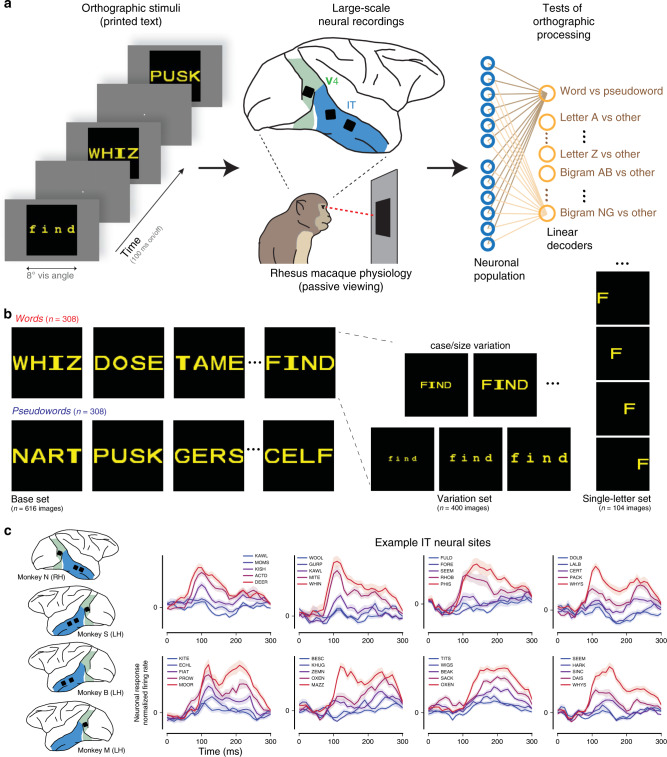
Conceptual schematic of experiment. **a** We recorded the activity of hundreds of neural sites in cortical area IT while monkeys passively viewed images of orthographic stimuli. (As a control, we also recorded from the dominant input to IT, area V4.) We then tested the sufficiency of the IT representation on 30 tests of orthographic processing (e.g., word classification, letter identification, etc.) using simple linear decoders. **b** Example visual stimuli. Images consisted of four-letter English words and pseudoword strings presented in canonical views, as well as with variation in case (upper/lower) and size (small/medium/large), and single letters presented at four different locations. **c** Responses of example IT neural sites. The approximate anatomical locations of implanted arrays are shown on the left for each monkey. Example neural activity in response to stimuli are shown for eight example neural sites in IT. Each trace corresponds to responses (mean over repetitions) to five example images chosen to illustrate the full range of evoked response; the color of each trace corresponds to the response magnitude, and is not indicative of whether a string corresponded to a word or pseudoword. Shaded areas correspond to SE of the mean response, over stimulus repetitions (*n* > 30 repetitions per image).

To quantitatively test this prediction of the “IT precursor” hypothesis, we first operationally defined a set of 30 orthographic identification and categorization tasks, such as identifying the presence of a specific letter or specific bigram within a letter string (invariant letter/bigram identification), or sorting out English words from pseudowords (word classification, also known in human psycholinguistics as the lexical decision task). Given that animals have no semantic knowledge of English words, ‘word classification’ here refers to a visual (rather than lexical) discrimination task, i.e., the ability to categorize letter strings as words or pseudowords on the basis of visual and/or orthographic features, generalizing to novel letter strings drawn from the same generative distributions. Specifically, we used the generative distribution of four-letter English words (hereafter referred to as “words”) and nonsense combinations of four letters with one vowel and three consonant letters (hereafter referred to as “pseudo-words”). We do not claim this set of tasks to be an exhaustive characterization of orthographic processing, but an unbiased starting point for that greater goal. We recorded the spiking activity of hundreds of neural sites in V4 and IT of rhesus macaque monkeys while they passively viewed images of letters, English words and pseudoword strings (Fig. 1a). We then asked whether adding a simple neural readout (biologically plausible linear decoders, cross-validated over letter strings) on top of the macaque IT representation could produce a neural substrate of orthographic processing. We found that linear decoders that learn from IT cortex activity easily achieved baboon-level performance on these tasks, and exhibited a pattern of behavioral performance that was highly correlated with the corresponding baboon behavioral pattern. These behavioral tests were also met by leading artificial neural network models of the non-human primate ventral stream, but not by low-level representations of those models. Taken together, these results show that, even in untrained non-human primates, the population of IT neurons contains an explicit (i.e., linearly separable), if still imperfect, representation of orthographic stimuli that might have been later “recycled” to support orthographic processing behaviors in higher primates such as humans.

## Results

### Tests of orthographic processing

Our primary goal was to experimentally test the capacity of neural representations in the primate ventral visual pathway to support orthographic classification tasks. To do so, we recorded the activity of hundreds of neurons from the top two levels of the ventral visual cortical hierarchy of rhesus macaque monkeys. Neurophysiological recordings were made in four Rhesus monkeys using chronically implanted intracortical microelectrode arrays (Utah) implanted in the inferior temporal (IT) cortex, the topmost stage of the macaque ventral visual stream hierarchy. As a control, we also collected data from upstream visual cortical area V4, which provides the dominant input to IT (Fig. 1a). The majority of the collected neurophysiological data corresponded to multi-unit spiking activity; hence we conservatively refer to these neural samples as neural sites. Neuronal responses were measured while each monkey passively viewed streams of images, consisting of alphabet letters, English words, and pseudoword strings, presented in a rapid serial visual presentation (RSVP) protocol at the center of gaze (Fig. 1). Crucially, monkeys had no previous supervised experience with orthographic stimuli, and they were not rewarded contingently on the stimuli, but solely for accurately fixating. This experimental procedure resulted in a large dataset of 510 IT neural sites (and 277 V4 neural sites) in response to up to 1120 images of orthographic stimuli.

To test the sufficiency of the IT representation for orthographic processing, we used simple linear decoders (as biologically plausible approximations of downstream neural computations, see Methods) to test each neuronal population on a battery of 30 visual orthographic processing tasks: 20 invariant letter identification tasks, eight invariant bigram identification tasks, and two variants of the word classification task. For each behavioral test, we used a linear decoder, which computes a simple weighted sum over the IT population activity, to discriminate between two classes of stimuli (e.g., words versus pseudowords). The decoder weights are learned using the IT population responses to a subset of stimuli (using 90% of the stimuli for training), and then the performance of the decoder is tested on held-out stimuli. The overarching prediction of the “IT precursor” hypothesis was that, if a putative neural mechanism (i.e., a particular readout of a particular neural population) is sufficient for primate orthographic processing behaviors, then, it should be easy to learn (i.e., few supervised examples), its learned performance should match the overall primate performance, and its learned performance should have similar patterns of errors as primates that have learned those same tasks. This logic has been previously applied to the domain of core object recognition to uncover specific neural linking hypotheses^25^ that have been successfully validated with direct causal perturbation of neural activity^26,27^.

### Word classification

We first focused on the visual discrimination of English words from pseudowords (word classification) using a random subset of the stimuli tested on baboons^17^. We collected the response of 510 IT neural sites and 277 V4 neural sites to a base set of 308 four-letter written words and 308 four-letter pseudowords (see Fig. 1b, base set for example stimuli). Figure 1c shows the stimulus-locked response of eight example neural sites in IT to five example strings chosen to illustrate the full range of response highlighted (dark curves). Each neural site was tested with all 616 stimuli in the base set, but each site’s response to only five are shown. These examples illustrate that IT sites reliably respond to letter strings above baseline, with greater response to some strings over others.

**Fig. 2:**
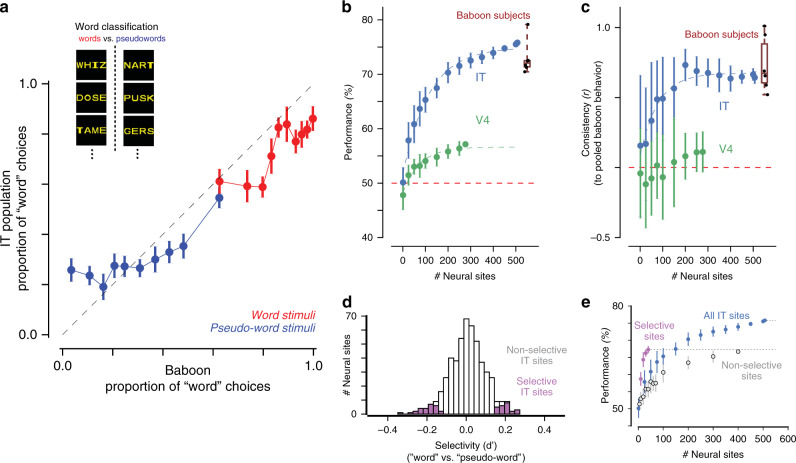
Sufficiency of IT population for word classification. **a** Comparison of baboon behavior and a linear readout of IT neurons, plotted as the proportion of stimuli categorized as “words.” The 616 individual stimuli were grouped into equally sized bins based on the baboon performance, separately for words (red) and pseudowords (blue). Error bars correspond to SEM, obtained via bootstrap resampling over stimuli (*n* = 100 bootstrap samples); dashed line corresponds to unity line, demarking a perfect match between baboon behavior and IT-based decoder outputs. **b** Performance of decoders trained on IT and V4 representations on word classification, for varying number of neural sites. Distribution of individual baboon performance is shown on the right (median 72%, interquartile range = 1%, data range = 8.7%, *n* = 6 baboon subjects). **c** Consistency with baboon behavioral patterns of decoders trained on IT and V4 representations, for varying number of neural sites. Distribution of individual baboon consistency is shown on the right (median $\tilde \rho = 0.67$, interquartile range = 0.27, data range = 0.49, *n* = 6 baboon subjects). **d** Distribution of selectivity of word classification for individual IT sites, highlighting the subpopulation of sites with selectivity significantly different from zero. **e** Performance of decoders trained on subpopulation of selective sites from **d** compared to remaining IT sites and all IT sites. Error bars in **b**, **c**, and **e** correspond to SD across samples of neural sites (*n* = 100 feature samples).

To test the capacity of the IT neural representation to support word classification, we trained a linear decoder using the IT population responses to a subset of words and pseudowords, and tested the performance of the decoder on held-out stimuli. This task requires generalization of a learned classification to novel orthographic stimuli, rather than the memorization of orthographic properties. Figure 2a shows the output choices of the linear readout of IT neurons, plotted as the probability of categorizing stimuli as words, as compared to behavioral choices of a pool of six baboons, as previously measured by Grainger et al.^17^. For ease of visualization, the 616 individual stimuli were grouped into equally sized bins based on the baboon performance, separately for words and pseudowords. We qualitatively observe a tight correspondence between the behavioral choices made by baboons and those measured by the linear decoder trained on the IT population. To quantify this similarity, we benchmarked both the overall performance (accuracy) and the consistency of pattern of errors of the IT population with respect to this previously measured median baboon behavior on the same images.

We first found that decoders trained on the IT population responses achieved high performance (76% for 510 neural sites) on word classification on new images, with about 250 randomly sampled IT neural sites matching the median performance of baboons doing this task (Fig. 2b, blue). Could any neural population achieve this performance? As a first control, we tested the upstream cortical area V4. We found that the tested sample of V4 neurons did not achieve high performance (57% for 277 V4 neural sites), failing to match baboon performance on this task (Fig. 2b, green).

We next tested whether baboons and neural populations exhibited similar behavioral patterns across stimuli, e.g., whether letter strings that were difficult to categorize for baboons were similarly difficult for these neural populations. To reliably measure behavioral patterns in each individual baboon subject, we grouped the 616 individual stimuli into equally sized bins based on an independent criterion (the average bigram frequency of each string in English, see Methods), separately for words and pseudowords. For both baboons and decoders, we then estimated the average unbiased performance for each stimulus bin using a sensitivity index (d’); this resulted in a ten-dimensional pattern of unbiased performances. We measured the similarity between patterns from a tested neural population and the pool of baboons using a noise-adjusted correlation (Methods). The pattern of performances obtained from the IT population was highly correlated with the corresponding baboon pool behavioral pattern (noise-adjusted correlation $\tilde \rho = 0.64$; Fig. 2c, blue). Could any neural population exhibit baboon-like behavioral patterns? On the contrary, we found that this correlation was significantly higher than the corresponding value estimated from the V4 population, which is only one visual processing stage away from IT ($\tilde \rho = 0.11$; Fig. 2c, green). By holding out data from each baboon subject from the pool, we additionally estimated the consistency between each individual baboon subject to the remaining pool of baboons (median $\tilde \rho = 0.67$, interquartile range = 0.27, *n* = 6 baboon subjects). This consistency value corresponds to an estimate of the ceiling of behavioral consistency, accounting for intersubject variability. Importantly, the consistency of IT-based decoders is within this baboon behavioral range; this demonstrates that that this neural mechanism is as consistent to the baboon pool as each individual baboon is to the baboon pool, at this behavioral resolution. Together, these results suggest that the distributed neural representation in macaque IT cortex is sufficient to explain the word classification behavior of baboons, which itself was previously found to be correlated with human behavior on the same four-letter strings^17^.

We next explored how the distributed IT population’s capacity for supporting word classification arose from single neural sites. Figure 2d shows the distribution of word selectivity of individual sites in units of d’, with positive values corresponding to increased firing rate response for words over pseudowords. Across the population, IT did not show strong selectivity for words over pseudowords (average d’ = 0.008 ± 0.09, mean, SD over 510 IT sites), and that no individual IT site was strongly selective for words vs. pseudowords (|d’| < 0.5 for all recorded sites). However, a small but significant proportion of sites (10%; *p* < 10^−5^, binomial test with 5% probability of success) exhibited a weak but significant selectivity for this contrast (inferred by a two-tailed exact test with bootstrap resampling over stimuli). This subset of neural sites includes both sites that responded preferentially to words and sites that responded preferentially to pseudowords. We measured the word classification performance of decoders trained on this subpopulation of neural sites, compared to the remaining subpopulation. Importantly, to avoid a selection bias in this procedure, we selected and tested neural sites based on independent sets of data (disjoint split-halves over trial repetitions). As shown in Fig. 2e, decoders trained on this subset of selective neural sites performed better than a corresponding sample from the remaining nonselective population, but not as well as decoders trained on the entire population, suggesting that the population’s capacity for supporting word classification relies heavily but not exclusively on this small subset of selective neural sites.

We next examined whether this subset of selective neural sites was topographically organized on the cortical tissue. For this subset of neural sites, we did not observe a significant hemispheric bias (*p* = 0.13, binomial test with probability of success matching our hemisphere sampling bias), or significant spatial clustering within each 10 × 10 electrode array (Moran’s I = 0.11, *p* = 0.70, see Methods). Thus, we observed no direct evidence for topographically organized specialization (e.g., orthographic category-selective domains) in untrained non-human primates, at the resolution of single neural sites. Taken together, these results suggest that word classification behavior could be supported by a sparse, distributed readout of the IT representation in untrained monkeys, and provide a baseline against which to compare future studies of trained monkeys.

### Tests of invariant orthographic processing

**Fig. 3:**
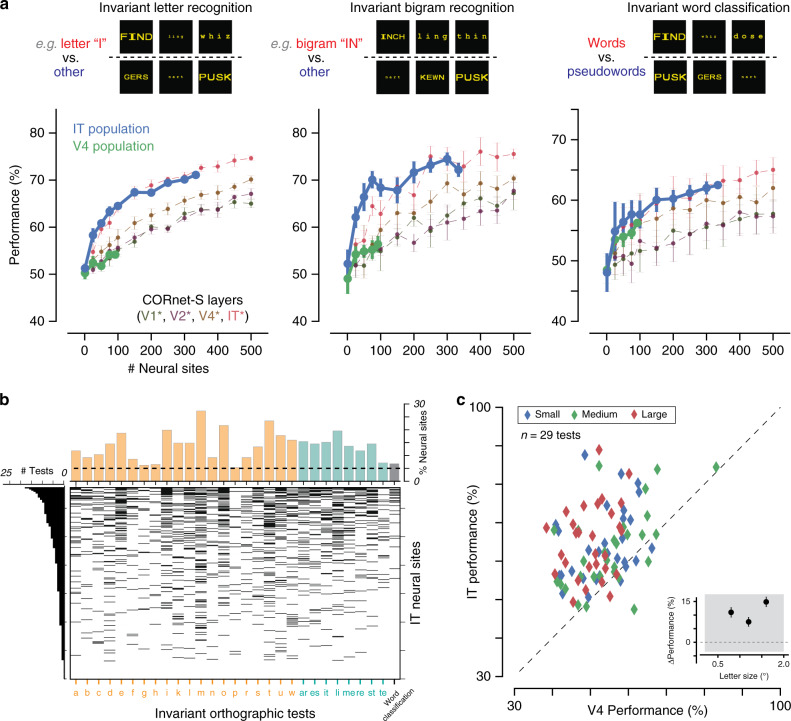
Performance of IT population on a battery of orthographic processing tasks. **a** Performance of linear decoders trained on the IT and V4 representations on invariant orthographic tests, grouped into letter identification (*n* = 20 tests), bigram identification (*n* = 8 tests) and invariant word classification. Performance of artificial representations sampled from layers of deep convolutional neural network model CORnet-S are shown in grey. Error bars correspond to SD across feature samples (neural sites or CORnet-S features). **b** Selectivity of individual IT sites over 29 invariant orthographic processing tests. The heatmap shows selectivity significantly different from zero overall pairs of neural sites and tests. The histogram above shows the number of behavioral tests (N_t_) that each neural site exhibited selectivity for; neural sites are ordered by increasing N_t_. The histogram on the right shows the proportion of neural sites exhibiting selectivity for each test; the behavioral tests are ordered alphabetically within each task group (letter identification in orange, bigram identification in cyan, and word classification in gray). Dashed line corresponds to proportion of tests expected from chance (alpha = 5%). **c** Comparison of V4 and IT linear decoder performance across all 29 invariant orthographic tests from **a** and **b**, separated by letter size (small, medium, large). Inset shows the average difference in performance between IT and V4 (mean ± SE, *n* = 29 tests; positive values corresponding to IT > V4), for each letter size. The shaded region corresponds to the “normal” range of letter sizes, over which humans exhibit substantial tolerance. IT linear decodes consistently outperform V4 across this range of stimulus sizes, suggesting that the observed differences between IT and V4 are not simply a consequence of stimulus size.

Human readers can not only discriminate between different orthographic objects, but also do so with remarkable tolerance to variability in printed text. For example, readers can effortlessly recognize letters and words varying in up to two orders of magnitude in size, and are remarkably tolerant to variations in printed font (e.g., upper vs lower case)^3,28^. To investigate such invariant orthographic processing behaviors, we measured IT-decoder performance for stimuli that vary in font size and font case, for a subsampled set of strings (40 words, 40 pseudowords, under five different variations for a total of 400 stimuli). We trained linear decoders on subsets of stimuli across all variations, and tested on held-out stimuli, for a total of 29 behavioral tests (20 invariant letter recognition tests, 8 invariant bigram recognition tests, and one test of invariant word classification). Figure 3a shows the performance of a decoder trained on the IT and V4 neuronal representations on each of these three types of behavioral tests, as a function of the neural sample size. The IT population achieved relatively high performance across all tasks, and that this performance was greater than the corresponding performance from the V4 population. We note that performance values for invariant word classification should not be directly compared with those in Fig. 2b, as invariant tests here were conducted with fewer training examples for the decoders.

We additionally tested the feature representation obtained from a deep recurrent convolutional neural network model of the ventral stream on the exact same behavioral tasks. Specifically, we tested the CORnet-S model^29^, as it is currently the best match to the primate ventral visual stream^30,31^ and provides an independently simulated estimate of the neuronal population responses from each retinotopically defined cortical area in the ventral visual hierarchy (V1, V2, V4, and IT). Figure 3 shows the performance of decoders trained on each simulated neuronal population on invariant letter identification, invariant bigram identification, and invariant word classification, as a function of the number of model units used for decoding. The last layer of CORnet-S (simulated IT) significantly outperforms earlier layers (simulated upstream areas V1, V2, and V4) on these invariant orthographic discrimination tasks, and tightly matches the performance of the actual IT population.

Next, we tested how the IT population’s capacity for these 29 invariant orthographic processing tests was distributed across individual IT neural sites. We computed the selectivity of individual sites in units of d’ for each of these tests, and estimated the statistical significance of each selectivity index using a two-tailed exact test with bootstrap resampling over stimuli (see Methods). Figure 3b shows a heatmap of significant selectivity indices for all pairs of neural sites and behavioral tests; each row corresponds to one behavioral test, each column to a single IT neural site, and filled bins indicate statistically significant selectivity. The histogram above shows the number of behavioral tests that each neural site exhibited selectivity for (median: three tests, interquartile range: 5), and the histogram on the right shows the proportion of neural sites exhibiting selectivity for each test (median: 49/337 neural sites, interquartile range: 23/337).

Finally, we tested whether the observed decoding performance difference between IT and V4 could simply reflect the stimulus size, such that V4 might outperform IT for smaller stimuli (e.g., because V4 neurons have smaller receptive fields than IT neurons). Recall that our stimulus set contained images with three letter sizes, where individual letters each spanned 0.8°, 1.2°, or 1.6° of visual angle; these letter sizes partially covers the “normal” range where human readers exhibit virtually no change in reading abilities (0.2°–2°)^32^. Taking advantage of this, we directly compared V4 and IT performance across all invariant orthographic tests, separating by letter size. Figure 3c shows this comparison, with most points above the unity line. The inset shows the difference in performance between IT and V4 (with positive values corresponding to IT > V4), for each letter size, averaged across the 29 behavioral tests; the shaded region corresponds to the “normal” range of letter sizes. IT populations consistently outperform V4 across the tested range of stimulus sizes, suggesting that the performance differences between IT and V4 decode performance do not simply reflect stimulus size.

Taken together, these results suggest that sparse, distributed read-outs of the adult IT representation of untrained non-human primates are sufficient to support many visual discrimination tasks, including ones in the domain of orthographic processing, and that the neural mechanisms corresponding to these read-outs could be learned with a small number of training examples (median: 48 stimuli; interquartile range: 59, *n* = 30 behavioral tests). Furthermore, this capacity is not captured by lower-level representations, including neural samples from the dominant visual input to IT (area V4) and low-level ventral stream representations as approximated by state-of-the-art artificial neural network models of the ventral stream.

### Encoding of orthographic stimuli

The availability of IT neuronal responses to orthographic stimuli allowed us to begin to address the question of how such stimuli are encoded at the single-neuron level. Behavioral and brain-imaging observations in human readers have led to several proposals concerning the putative neural mechanisms underlying human orthographic abilities. For example, the local combination detector (LCD) hypothesis posits a hierarchy of cortical representations whereby neurons encode printed letter strings at increasing scale and complexity, from tuning to simple edges and letters to intermediate combinations of letters (e.g., letter bigrams) and finally to complex words and morphemes over the cortical hierarchy^2^. Other theories have proposed that letter position information is encoded in the precise timing of spikes^33,34^. To date, it has been difficult to directly test such hypotheses. Here, to help constrain the space of encoding hypotheses, we characterized the response properties of hundreds of individual IT neural sites to words and to their component letters.

**Fig. 4:**
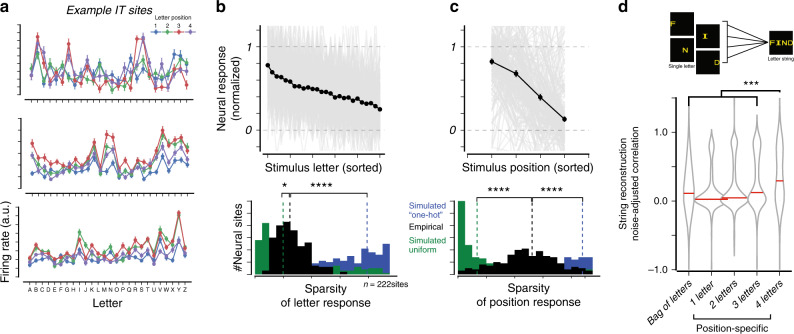
Encoding properties of single IT sites. **a** Firing rate responses to individual letter stimuli (26 letters at four positions) for three example IT sites. Error bars correspond to SEM across stimulus repetitions (*n* > 30 repetitions per image). **b** (top) Gray lines indicate the normalized mean response to each of the 26 letters, sorted (using an independent half of the data) for each of 222 IT neural sites. The black line indicates the population average (mean ± SE across sites, *n* = 222 sites; note that SE is very small). Neural sites reliably respond more to some letters than others. However, this modulation is not very selective to individual letters or small numbers of letters, as quantified by the sparsity of letter responses (bottom panel). **c** Individual sites were also modulated by the letter position, exhibiting tolerance across positions (formatted as in **b**). **d** To test if the encoding of letter strings can be approximated as a local combination of responses to individual letters, we reconstructed letter string responses from letter responses (using linear regressions, cross-validating over letter strings), for each neural site. The violin plots show the distribution of noise-adjusted correlation of different regression models (*n* = 222 neural sites); the red line corresponds to the median of each distribution. The “bag of letters” model uses responses of each of the four letters, at arbitrary positions, as predictors. Each of the position-specific models uses the responses of up to four letters at the appropriate position as predictors. Across all panels, asterisks are defined as: *p < 0.05, **p < 0.01, ***p < 0.001, ****p < 0.0001 obtained by permutation test of median distributions.

We first asked if individual IT neural sites exhibit any selectivity for letters, i.e., if firing rates reliably differ for different letters. To test this, we measured the selectivity of IT responses to each of the 26 alphabet letters, each presented at four different retinal positions. Figure 4a shows the “tuning curve” for three example IT neural sites. Consistent with the known image selectivity and position tolerance of IT neurons^19,22,23^, the responses of these IT neural sites were significantly modulated by both letter identity and letter position, with each example site responding to some but not all individual letters.

We focused on 222 (out of 338) neural sites with reliable response patterns across the single letter stimulus set (*p* < 0.01, significant Pearson correlation across split-halves over repetitions). Figure 4b (top) shows the average normalized response to each of the 26 letters, across these 222 neural sites. For each neural site, letters were sorted according to the site’s response magnitude, estimated using half of the data (split-half of stimulus repetitions) to ensure statistical independence; we then plotted the sorted letter response measured on the remaining half (individual sites in gray, mean ± SEM in black). Across the entire population, some neural sites reliably respond more to some letters than others, but this modulation is generally not selective for one or a small number of letters. Rather, sites tended to respond to a broad range of letters, as quantified by the sparsity of letter responses (sparsity index *SI* = 0.24 ± 0.01; median ± standard error of median; Fig. 4b, bottom panel, black bars). To provide references for this empirical SI distribution, we estimated the corresponding SI distributions for two extreme simulated hypotheses: whereby (1) neural sites respond equally to all letters (uniform hypothesis), and (2) neural sites respond to only one letter (one-hot hypothesis), obtained via random permutations of our data (see Methods). As shown in Fig. 4b, the empirically observed median sparsity was significantly greater than predicted by the uniform hypothesis (*p* = 0.02, permutation test of median distributions), and significantly less than expected by the one-hot hypothesis (*p* < 0.0001, permutation test of median distributions). We repeated this analysis for letter positions: individual sites were also modulated by letter position (Fig. 4c, formatted as in Fig. 4b), with a greater response to letters presented contralateral to the recording site, while also exhibiting substantial tolerance across positions. The empirically observed distribution of sparsity of responses across positions (*SI* = 0.52 ± 0.02; median ± standard error of median) was significantly different from both simulated uniform and one-hot hypotheses (*p* < 0.0001, permutation test of median distributions).

Next, we asked whether the encoding of letter strings could be approximated as a sum of responses to individual letters. To test this, we linearly regressed each site’s response to letter strings on the responses to the corresponding individual letters at the corresponding position, cross-validating over letter strings. Using the neural responses to all four letters, the predicted responses of such a linear reconstruction were modestly correlated with the measured responses to letter strings (see Fig. 4d, rightmost distribution; $\tilde \rho = 0.29 \pm 0.06$, median ± standard error of median, *n* = 222 neural sites). To investigate if this explanatory power arose from all four letters, or whether 4-letter string responses could be explained just as well by a substring of letters, we trained and tested linear regressions using responses to only some (1, 2, or 3) letters. Given that there are many possible combinations for each, we selected the best such mapping from the training data, ensuring that selection and testing were statistically independent. Reconstructions using only some of the letters were poorer than those using all four letters in predicting letter string responses (three letters: $\tilde \rho = 0.12 \pm 0.02$, median ± standard error of median), and this difference was statistically significant (*p* = 0.002, one-tailed exact test on distributions of medians obtained by bootstrap resampling over neural sites). Finally, we tested how well a position-agnostic (or “bag of letters”) model performed on the same reconstruction task by trained and test linear regressions that mapped responses of letters, with the incorrect position (using a fixed, random shuffling of letter positions) on reconstructing the responses to whole letter strings. We found that this “bag of letters” model performed significantly worse ($\tilde \rho = 0.11 \pm 0.02$, median ± standard error of median, *p* = 0.0004, one-tailed exact test on distributions of medians obtained by bootstrap resampling over neural sites).

All correlation values reported above were adjusted to account for the reliability of measured neural responses, such that a fully predictive model would have a distribution of estimated noise-adjusted correlations overlapping with 1.0 regardless of the finite amount of data that were collected. Yet, across all tested neural sites, the maximal value of $\tilde \rho = 0.29$ that we obtained using the linear superposition of position-specific responses to the four letters was substantially lower than 1.0. Thus, the pure summation of neural responses to individual letter identity and position explained only a small part of the reliable neural responses to four-letter strings, suggesting that nonlinear responses to local combinations of letters were also present. Future work using stimuli comprising a larger number of letter combinations can explore to what extent IT neural sites respond, for instance, to specific letter bigrams, as predicted by some models^2,35^, or to other complex invariant visual features.

### Mirror-symmetric tuning

**Fig. 5:**
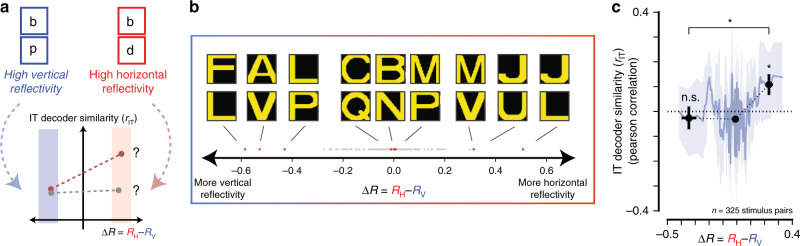
Test of mirror symmetry in IT. **a** If the left-right inversion errors of early readers are due in part to the high-level visual representation of letters, this predicts that the IT population decodes should be more similar (i.e., more likely to be confused) for pairs of letters that have high horizontal reflectivity (e.g., “b” and “d”), compared to pairs of letter than have high vertical reflectivity (e.g. “b” and “p”). In other words, we predict *r*_*IT*_ to be greater for positive ΔR than for negative ΔR (bottom, red curve). **b** We quantified the amount of horizontal and vertical mirror symmetry between all 325 pairs of letters in our stimulus set, and sorted them along the dimensions of ΔR (see text). The gray dots show the corresponding distributions for each, with nine example stimulus pairs (red dots) selected to span the entire range. **c** The dependence of IT-decoder representational similarity *r*_*IT*_ to the empirically measured ΔR, overall 325 letter pairs. The smooth blue line shows the rolling average using a boxcar window of five stimulus pairs. We binned ΔR into three bins spanning three equal ΔR ranges; the black markers show the average *r*_*IT*_ for each bin (mean ± SE over stimulus pairs). We observe a positive average *r*_*IT*_ for the rightmost bin, but not for the left-most; the difference between these two measurements was modest but significant. Asterisk corresponds to *p* < 0.05 obtained by unpaired one-tailed two-sample *t*-test.

We next examined the response patterns of each IT site across different letter stimuli. Prior neurophysiological work has shown that a small proportion of neurons in IT exhibit “mirror-symmetric” tuning^36–38^, i.e., respond similarly to stimuli that are horizontal mirror images of one another, but not to corresponding vertical mirror-image pairs. This phenomenon has been hypothesized to underlie the left-right inversion errors of children learning to read, but has not been directly tested. In light of this, we sought to test whether the output patterns of decoders trained on the recorded IT population exhibited any evidence of mirror symmetry in response to orthographic stimuli. Figure 5a outlines the logic of our analysis: if the left-right inversion errors of early readers is due in part to the high-level visual representation of letters, then we should find IT population decodes to be more similar (i.e., more likely to be confused) for pairs of letters that have high horizontal reflectivity (e.g., b and d), compared to pairs of letter than have high vertical reflectivity (e.g., b and p). To test this, we quantified the amount of horizontal and vertical reflectivity, *R*_*H*_ and *R*_*H*_, respectively, between all 325 pairs of letters in our stimulus set (see Methods). Next, we measured the similarity *r*_*IT*_ of this stimulus pair with respect to an IT-based decoder as the Pearson correlation between the corresponding decoder outputs (see Methods). Finally, across many such stimulus pairs, we relate the relative horizontal reflectivity ΔR = *R*_*H*_ − *R*_*V*_ to the IT-decoder similarity *r*_*IT*_ (Fig. 5a, bottom). If IT-based decoders exhibit a tendency toward (horizontal) “mirror symmetric” confusion, we expect *r*_*IT*_ to be greater for positive ΔR than for negative ΔR (see Fig. 5a, bottom panel, red curve). Alternatively, we expect no dependence of *r*_*IT*_ on ΔR (see Fig. 5a, bottom panel, gray curve).

While our stimulus set did not include any image pairs that are perfect horizontal or vertical mirror-images images of one another, we nevertheless attempted to relate the similarity in IT of specific stimulus pairs to their empirical pixel-level reflectivity. Figure 5b shows the distributions of ΔR = *R*_*H*_ − *R*_*V*_ over 325 unique pairs of letters in our stimulus (gray dots), with nine example stimulus pairs (red dots) selected to illustrate the entire range. ΔR is large for pairs of approximately horizontally symmetric letters (J and L), but not pairs with overall pixel overlap (e.g., C and Q) or pairs of approximately vertically symmetric letters (e.g., A and V). Importantly, we did not consider identity pairs (a letter and itself) to avoid inflating our results by bias in horizontal symmetry present in alphabet letters (see Methods). Figure 5c shows the dependence of the IT-decoder representational similarity *r*_*IT*_ to the empirically measured ΔR overall 325 unique pairs of letters. The smooth blue line shows the rolling average overall 325 letter pairs, averaging over a boxcar window of five letter pairs. To obtain summary statistics from these data, we binned ΔR into three bins spanning equal ΔR sizes. We observed a positive average *r*_*IT*_ for the rightmost bin (*r*_*IT*_ = 0.11 ± 0.04, *p* = 0.01; one-tailed *t*-test), but not for the left-most bin (*r*_*IT*_ = −0.03 ± 0.05, *p* = 0.30; one-tailed *t*-test); the difference between these two measurements was modest but significant (*p* = 0.025, unpaired one-tailed two-sample *t*-test). These results provide modest evidence that a readout of the IT representation of letters is consistent with previously reported left-right mirror symmetry, and suggest that a targeted experiment designed to directly test this hypothesis is warranted.

Taken together, these observations demonstrate that individual IT neural sites in untrained non-human primates, while failing to exhibit strong orthographic specialization, collectively suffice to support a battery of orthographic tasks. Importantly, these observations establish a number of relevant quantitative baselines, a preregistered benchmark to which future studies of the ventral stream representations in monkeys trained on orthographic discriminations, or in literate humans, could be directly compared to.

## Discussion

A key goal of human cognitive neuroscience is to understand how the human brain supports the ability to learn to recognize written letters and words. This question has been investigated for several decades using human neuroimaging techniques, yielding putative brain regions that may uniquely underlie orthographic abilities^7–9^. In the work presented here, we sought to investigate this issue in the primate ventral visual stream of naïve rhesus macaque monkeys. Non-human primates such as rhesus macaque monkeys have been essential to study the neuronal mechanisms underlying human visual processing, especially in the domain of object recognition where monkeys and humans exhibit remarkably similar behavior and underlying brain mechanisms, both neuroanatomical and functional^13–16,39,40^. Given this strong homology, and the relative recency of reading abilities in the human species, we reasoned that the high-level visual representations in the primate ventral visual stream could serve as a precursor that is recycled by developmental experience for human orthographic processing abilities. In other words, we hypothesized that the neural representations that directly underlie human orthographic processing abilities are strongly constrained by the prior evolution of the primate visual cortex, such that representations present in naïve, illiterate, non-human primates could be minimally adapted to support orthographic processing. Here, we observed that orthographic information was explicitly encoded in sampled populations of spatially distributed IT neurons in naïve, illiterate, non-human primates. Our results are consistent with the hypothesis that the population of IT neurons in each subject forms an explicit (i.e., linearly separable, as per ref. ^21^) representation of orthographic objects, and could serve as a common substrate for learning many visual discrimination tasks, including ones in the domain of orthographic processing.

We tested a battery of 30 orthographic tests, focusing on a word classification task (separating English words from pseudowords). This task is referred to as “lexical decision” when tested on literate subjects recognizing previously learned words (i.e., when referencing a learned lexicon). For nonliterate subjects (e.g., baboons or untrained IT decoders), word classification is the ability to identify orthographic features that distinguish between words and pseudowords and generalize to novel strings. This generalization must rely on specific visual features whose distribution differs between words and pseudowords; previous work suggests that such features may correspond to specific bigrams^17^, position-specific letter combinations^41^, or distributed visual features^42^. While this battery of tasks is not an exhaustive characterization of orthographic processing, we found that it has the power to distinguish between alternative hypotheses. Indeed, these tasks could not be accurately performed by linear readout decoders of the predominant input visual representation to IT (area V4) or by approximations of lower levels of the ventral visual stream, unlike many other coarse discrimination tasks (e.g., contrasting orthographic and nonorthographic stimuli). We note that the successful classifications from IT-based decoders do not necessarily imply that the brain exclusively uses IT or the same coding schemes and algorithms that we have used for decoding. Rather, the existence of this sufficient code in untrained and illiterate non-human primates suggests that the primate ventral visual stream could be minimally adapted through experience-dependent plasticity to support orthographic processing behaviors.

These results are consistent with a variant of the “neuronal recycling” theory, which posits that the features that support visual object recognition may have been coopted for written word recognition^5,6,24^. Specifically, this variant of the theory is that humans have inherited a pre-existing brain system (here, the ventral visual stream) from recent evolutionary ancestors, and they either inherited or evolved learning mechanisms that enable individuals to adapt the outputs of that system during their lifespan for word recognition and other core aspects of orthographic processing. Consistent with this, our results suggest that prereading children likely have a neural population representation that can readily be reused to learn invariant word recognition. Relatedly, it has been previously proposed that the initial properties of this system may explain the child’s early competence and errors in letter recognition, e.g., explaining why children tend to make left-right inversion errors by the finding that IT neurons tend to respond similarly to horizontal mirror images of objects^36,37,43^. Consistent with this, we here found that the representation of IT-based decoders exhibited a similar signature of left-right mirror symmetry. According to this proposal, this neural representation would become progressively shaped to support written word recognition in a specific script over the course of reading acquisition, and may also explain why all human writing systems throughout the world rely on a universal repertoire of basic shapes^24^. As shown in the present work, those visual features are already well encoded in the ventral visual pathway of illiterate primates, and may bias cultural evolution by determining which scripts are more easily recognizable and learnable.

A similar “neuronal recycling hypothesis” has been proposed for the number system: all primates may have inherited a pre-existing brain system (in the intraparietal sulcus) in which approximate number and other quantitative information is well encoded^44,45^. It has been suggested that these existing representations of numerosity may be adapted to support exact, symbolic arithmetic, and may bias the cultural evolution of numerical symbols^6,46^. Likewise, such representations have been found to spontaneously emerge in neural network models optimized for other visual functions^47^. Critically, the term “recycling,” in the narrow sense in which it was introduced, refers to such adaptations of neural mechanisms evolved for evolutionary older functions to support newer cultural functions, where the original function is not entirely lost and the underlying neural functionality constrains what the brain can most easily learn. It remains to be seen whether all instances of developmental plasticity meet this definition, or whether learning may also simply replace unused functions without recycling them^48^.

In addition to testing a prediction of this neuronal recycling hypothesis, we also explored the question of how orthographic stimuli are encoded in IT neurons. Decades of research has shown that IT neurons exhibit selectivity for complex visual features with remarkable tolerance to changes in viewing conditions (e.g., position, scale, and pose)^19,22,23^. More recent work demonstrates that the encoding properties of IT neurons, in both humans and monkeys, is best explained by the distributed complex invariant visual features of hierarchical convolutional neural network models^30,49,50^. Consistent with this prior work, we here found that the firing rate responses of individual neural sites in macaque IT was modulated by, but did not exhibit strong selectivity to orthographic properties, such as letters and letter positions. In other words, we did not observe precise tuning as postulated by “letter detector” neurons, but instead coarse tuning for both letter identity and position. It is possible that, over the course of learning to read, experience-dependent plasticity could fine-tune the representation of IT to reflect the statistics of printed words (e.g., single-neuron tuning for individual letters or bigrams). Moreover, such experience could alter the topographic organization to exhibit millimeter-scale spatial clusters that preferentially respond to orthographic stimuli, as have been shown in juvenile animals in the context of symbol and face recognition behaviors^18,51^. Together, such putative representational and topographic changes could induce a reorientation of cortical maps towards letters at the expense of other visual object categories, eventually resulting in the specialization observed in the human visual word form area (VWFA). However, our results demonstrate that, even prior to such putative changes, the initial state of IT in untrained monkeys has the capacity to support many learned orthographic discriminations.

In summary, we found that the neural population representation in IT cortex in untrained macaque monkeys is largely able, with some supervised instruction, to extract explicit representations of written letters and words. This did not have to be so—the visual representations that underlie orthographic processing could instead be largely determined over postnatal development by the experience of learning to read. In that case, the IT representation measured in untrained monkeys (or even in illiterate humans) would likely not exhibit the ability to act as a precursor of orthographic processing. Likewise, orthographic processing abilities could have been critically dependent on other brain regions, such as speech and linguistic representations, or putative flexible domain-general learning systems, that evolved well after the evolutionary divergence of humans and Old-World monkeys. Instead, we here report evidence for a precursor of orthographic processing in untrained monkeys. This finding is consistent with the hypothesis that learning rests on pre-existing neural representations which it only partially reshapes.

## Methods

### Subjects

The non-human subjects in our experiments were four adult male rhesus macaque monkeys (Macaca mulatta, subjects N, B, S, M). Surgical procedures, behavioral training, and neural data collection are described in detail below. All procedures were performed in compliance with the guideline of National Institutes of Health and the American Physiological Society, and approved by the MIT Committee on Animal Care.

### Visual īmages

We randomly subsampled 616 strings (308 words, 308 pseudowords) from the stimulus set used to test orthographic processing abilities in baboons by Grainger et al. Word strings consisted of four-letter English words, whereas pseudoword strings consisted of nonsense combinations of four letters, with one vowel and three consonant letters. The entire set of pseudowords contained bigrams that ranged from those that are very common in the English language (e.g., TH) to those that are very uncommon (e.g., FQ), as quantified by a broad distribution of English bigram frequency (median = 95, interquartile range = 1366; in units of count per million). As such, given the previously established link between bigram frequency and difficulty in word classification^17^, orthographic stimuli spanned a range of difficulties for the word vs pseudoword word classification task. From these 616 strings, we then generated images of these strings under different variations of generative parameters in font size (small/medium/large size) and font case (upper/lower case), fixing the font type (monotype), color (yellow), thus creating a total of 3696 images. We additionally generated images of individual alphabet letters at each of the possibly locations (26 letters × 4 locations × 6 variations in font case/size). We measured IT and V4 responses from passively fixating rhesus macaque monkeys (see below) for a subset of 1120 images from this stimulus set, and used previously measured behavior from trained baboons from the study by Grainger and colleagues^17^. Visual images were presented to span 8° of visual angle, matching our prior neurophysiological experiments. For small, medium and large stimulus variations, each individual letter spanned approximately 0.8°, 1.2°, and 1.6° of visual angle, and each four-letter string spanned 3.2°, 4.8°, and 6.4° of visual angle (i.e., the spacing between letters was proportional to the letter sizes themselves). Such letter sizes are within the range of 0.2°–2° where human readers exhibit virtually no change in reading abilities^32^. We did not test even smaller letter sizes, due to difficulties in measuring reliable neurophysiological signals with subdegree precision in awake and behaving monkeys (or humans), given the variability in gaze fixation, fixational eye movements, and gaze tracking.

### Baboon behavior

Baboon behavioral data from six guinea baboons performing a word classification task was obtained from prior work^17^. We focused our analysis on the aforementioned subsampled stimulus set (616 strings).

### Surgical implant of chronic microelectrode arrays

We surgically implanted each monkey with a head post under aseptic conditions. After behavioral training, we implanted multiple 10 × 10 microelectrode arrays (Utah arrays; Blackrock Microsystems) in V4 and IT cortex of each monkey. A total of 96 electrodes were connected per array. Each electrode was 1.5 mm long and the distance between adjacent electrodes was 400 μm. Array placements were guided by the sulcus pattern, which was visible during surgery. Approximate array locations for each monkey are shown in Fig. 1c. The electrodes were accessed through a percutaneous connector that allowed simultaneous recording from all 96 electrodes from each array. All behavioral training and testing were performed using standard operant conditioning (fluid reward), head stabilization, and real-time video eye tracking.

### Eye tracking

We monitored eye movements using video eye tracking (SR Research EyeLink 1000). Using operant conditioning and water reward, our subjects were trained to fixate a central white square (0.2°) within a square fixation window that ranged from ±2°. At the start of each behavioral session, monkeys performed an eye-tracking calibration task by making a saccade to a range of spatial targets and maintaining fixation for 500 ms. Calibration was repeated if drift was noticed over the course of the session.

### Electrophysiological recording

During each recording session, band-pass filtered (0.1 Hz–10 kHz) neural activity was recorded continuously at a sampling rate of 20 kHz using Intan Recording Controller (Intan Technologies, LLC). The majority of the data presented here were based on multi-unit activity, hence we refer to neural sites. We detected the multi-unit spikes after the raw data were collected. A multi-unit spike event was defined as the threshold crossing when voltage (falling edge) deviated by less than three times the standard deviation of the raw voltage values. In this manner, we collected neural data from macaque V4 and IT from four male adult monkeys (N, B, S, M, weighing between 7 and 10 kg) in a piecewise manner. We focused our analyses on neural sites that exhibited significant visual drive (determined by *p* < 0.001 comparing baseline activity to visually driven activity); this resulted in 510 IT neural sites and 277 V4 neural sites. Our array placements allowed us to sample neural sites from different parts of IT, along the posterior to anterior axis. However, we did not consider the specific spatial location of the site, and treated each site as a random sample from a pooled IT population. For each neural site, we estimated the repetition-averaged firing rate response in two temporal windows (70–170 ms and 170–270 ms after stimulus onset) and concatenated these firing rates for decoding analyses. Single-unit analyses focused on the 70–170 ms time interval.

### Linear decoders

To test the capacity of ventral stream neural representations to support orthographic processing tasks, we used linear decoders to discriminate between two classes of stimuli (e.g., words versus pseudowords) using the firing rate responses of neural populations. We used binary logistic regression classifiers with ten-fold cross-validation: decoder weights were learned using the neural population responses to 90% of stimuli and then the performance of the decoder is tested on held-out 10% of stimuli, repeating 10 times to test each stimulus. We repeated this process 10 times with random sampling of neurons. This procedure produces an output class probability for each tested stimulus, and we took the maximum of those as the behavioral choice of the decoded neural population. We use such linear classifier as simple biologically plausible models of downstream neuronal computations. Indeed, the trained linear decoder perform binary classifications by computing weighted sums of IT responses followed by a decision boundary, analogous to synaptic strengths and spiking thresholds of neurons downstream of IT.

### Deep neural network model behavior

We additionally tested a deep neural network model of the primate ventral stream on the exact same images and tasks. We used CORnet-S, a deep recurrent convolutional neural network model that has recently been shown to best match the computations of the primate ventral visual stream^29,31^. CORnet-S approximates the hierarchical structure of the ventral stream, with four areas each mapped to the four retinotopically defined cortical area in the ventral visual hierarchy (V1, V2, V4, and IT). To do so, we first extracted features from each CORnet-S layer on the same images. As with neural features, we trained back-end binary logistic regression classifiers to determine the ten-fold cross-validated output class probability for each image and for each label.

### Behavioral metrics

For each behavioral test, we measured the average unbiased performance (or balanced accuracy) as ${\mathrm{acc}} = \frac{{{\mathrm{HR}} + (1 - {\mathrm{FAR}})}}{2}$, where HR and FAR correspond to the hit-rate and false-alarm-rate across all stimuli.

For the word classification task, we additionally estimated behavioral patterns across stimuli. To reliably measure behavioral patterns in each individual baboon subject, we grouped the 616 individual stimuli into ten equally sized bins separately for words and pseudowords; bins were defined based on the average bigram frequency of each string in English. We then estimated the average unbiased performance for each stimulus bin using a sensitivity index: *d*′ = *Z*(*HR*) − *Z*(*FAR*)^52^, where HR and FAR correspond to the hit-rate and false-alarm-rate across all stimuli within the bin. Across stimulus bins, this resulted in a ten-dimensional pattern of unbiased performances.

### Behavioral consistency

To quantify the behavioral similarity between baboons and candidate visual systems (both neural and artificial) with respect to the pattern of unbiased performance described above, we used a measure called consistency ($\tilde \rho$) as previously defined^53^, computed as a noise-adjusted correlation of behavioral signatures^54^. For each system, we randomly split all behavioral trials into two equal halves and estimated the pattern of unbiased performance on each half, resulting in two independent estimates of the system’s behavioral signature. We took the Pearson correlation between these two estimates of the behavioral signature as a measure of the reliability of that behavioral signature given the amount of data collected, i.e., the split-half internal reliability. To estimate the consistency, we computed the Pearson correlation overall the independent estimates of the behavioral signature from the model (**m**) and the primate (**p**), and we then divide that raw Pearson correlation by the geometric mean of the split-half internal reliability of the same behavioral signature measured for each system: $\tilde \rho ({\boldsymbol{m}},{\boldsymbol{p}}) = \frac{{\rho ({\boldsymbol{m}},{\boldsymbol{p}})}}{{\sqrt {\rho ({\boldsymbol{m}},{\boldsymbol{m}})\rho (p,{\boldsymbol{p}})} }}$. Since all correlations in the numerator and denominator were computed using the same number of trials, we did not need to make use of any prediction formulas (e.g., extrapolation to larger number of trials using Spearman–Brown prediction formula). This procedure was repeated 10 times with different random split-halves of trials. Our rationale for using a reliability-adjusted correlation measure for consistency was to account for variance in the behavioral signatures that is not replicable by the experimental condition (image and task).

### Single-neuron analyses

For each neural site, we estimated the selectivity with respect to a number of contrasts (e.g., word vs pseudoword) using a sensitivity index: $d_{x,y}^\prime = \frac{{\mu _x - \mu _y}}{{\sqrt {\frac{1}{2}(\sigma _x^2 + \sigma _y^2)} }}$^52^. We obtained uncertainty estimates for single-neuron selectivity indices by bootstrap resampling over stimuli, and inferred statistical significance using two-tailed exact tests on the bootstrapped distributions.

We determined whether neural sites that exhibited significant selectivity for word classifications were topographically organized across the cortical tissue using Moran’s *I*^55^, a metric of spatial autocorrelation. We compared the empirically measured autocorrelation (averaged over six-electrode arrays) to the corresponding distributions expected by chance, obtained by shuffling each electrode’s selectivity 100 times.

We quantified the sparsity of neural responses to letter identity and position using a sparsity index SI^56^ as follows: $A\left( x \right) = \frac{{E[x]^2}}{{E[x^2]}}$, ${\mathrm{SI}}\left( x \right) = \frac{{1-A(x)}}{{1-1/N}}$, where E[.] denotes the expectation of, and *N* is the length of the vector *x*. In the absence of measurement noise, the SI has a value of 0 for a perfectly uniform response pattern, and a value of 1 for a perfectly one-hot response pattern. However, to estimate the expected SI values for uniform and one-hot conditions in the presence of measurement noise, we performed the following simulation. To simulate the uniform condition, we randomly shuffled the stimulus category on each stimulus repetition, and averaged this shuffled response vector across repetitions. This procedure estimates the expected repetition-average response for a neuron that responds uniformly across all stimuli, while fixing the variability across repetitions. To simulate the one-hot condition, we used half of the repetitions to infer the top responsive stimulus category, and shuffled all this stimulus category on the second split-half of the data. Averaging this across split-halves, we obtained the expected repetition-averaged one-hot response, fixing the variability across repetitions.

### Mirror-symmetry analyses

We quantified the horizontal and vertical reflectivity (R_H_, R_V_) of different letter pairs using a pixel overlap metric, defined as the ratio of intersection and union of pixels between two letters. Specifically, we measured the pixel overlap after applying a horizontal (or vertical) reflection on one letter (about the center of its bounding box to measure the amount of horizontal (or vertical) mirror symmetry. For each letter pair, we then estimated the difference ΔR = R_H_ − R_V_. We observed that the set of 26 alphabet letters are biased to have high ΔR, i.e., most alphabet letters are more horizontally symmetric than vertically symmetric to themselves. Given this bias, we did not include identity pairs (a letter and itself) in our analyses to avoid inflating the evidence for horizontal mirror symmetry. To measure the similarity of each stimulus pair with respect to IT-based decoders, we trained a multinomial logistic regression decoder on the IT population to identify individual letters, and used this learned decoder to map the IT representation into a 26-dimensional representation (with one dimension for each of the 26 alphabet letters). We measured the IT-decoder similarity for each pair of letter images via a Pearson correlation of the two 26-dimensional vectors corresponding to the pair at one position, and then averaged the similarity over the four possible positions, resulting in a pattern of IT-decoder similarity overall 325 unique pairs of letters (termed *r*_*IT*_).

## Supplementary information

Peer Review File

## Data Availability

The images used in this study and the behavioral and neural data will be available from a public repository (https://github.com/brain-score/brainio_collection).

## Code availability

The code to generate the associated figures will be available on a public GitHub repository (https://github.com/RishiRajalingham/NatureComms2020).
