# Supplementary material for: The inferior temporal cortex is a potential cortical precursor of orthographic processing in untrained monkeys
Source: Nat Commun. 2020 Aug 4;11:3886. doi: 10.1038/s41467-020-17714-3 (PMC7403350; doi:10.1038/s41467-020-17714-3)
Supplement: Supplementary file 1 — Peer Review File [file 41467_2020_17714_MOESM1_ESM.docx]

Peer Review Information

**Journal:** Nature Communications

**Manuscript title:** The inferior temporal cortex is a potential cortical precursor of orthographic processing in untrained monkeys

**Corresponding author name(s):** Dr James DiCarlo

**Editorial notes:**

**none**

**Reviewer comments & decisions:**

**Reviewer comments, first version:**

Reviewer #1 (Remarks to the Author):

The authors recorded neural representations in the primate ventral visual pathway to explore whether such signals would support orthographic classification tasks. Neuronal responses were measured from IT and V4 while each monkey passively viewed rapid streams of images, consisting of alphabet letters, English words, and pseudo-word strings, at the center of gaze. The authors found that IT-based decoders achieved baboon-level performance on these tasks, with a pattern of errors highly correlated to the previously reported primate behavior. This orthographic capacity was also present in the high-layer units of state-of-the-art artificial neural network models of the ventral stream.

This is a great study with fascinating data, and a pleasure to read. The study is a rare and most welcome combination of neuron recordings in monkeys, state-of-the-art decoder analyses and deep network modeling, and comparison with previously published behavioral data from baboons. The results for the first time demonstrate that the nescient state of IT in untrained monkeys has the capacity to support many learned orthographic discriminations characteristic for literate humans. As such, it is a very significant study about the neural precursors of literacy. I only have a few comments I would ask the authors to address prior to publication.

Major comments:
1. Abstract. I think the abstract is badly selling the results. Only two sentences are devoted to the actual findings, while the findings would deserve to be the main part of the abstract. A reader of the abstract would benefit from more information about the actual findings (and less background information).

2. Presentation of original data. In the results, the paper right away starts with the presentation of the decoder performance based on neuronal populations. While reading the results, I always wondered about the original neuronal data in the form of histograms, firing rates, or tuning curves. After all, this is what the decoder is working with. Later in Fig2 D,E, the paper speaks about the importance of neuronal selectivity, but again selectivity is presented in a quite derived way. Much later in the results, in the very last chapter, three tuning curves to letters are finally shown (Fig. 4A). While I appreciate this last chapter a lot, I think a reader would benefit from introducing original responses of neurons right at the beginning of the results in order to understand the gist of the derived population measures. I recommend this even if there would be slight redundancy with the last chapter.

3. Test for mirror-image confusion. Humans and animals confuse lateral mirror images, such as the letters “b” and “d”. Previous recordings in macaque IT cortex by Rollhagen & Olson (2000, Science) showed that neurons responded more similarly to members of a lateral mirror-image pair than to members of a vertical mirror-image pair. Similarly, Baylis and Driver (2001, NN) showed that shape-coding cells in the inferotemporal cortex of the monkey generalize between mirror images. It would be fascinating to know whether mirror-image confusion occurred in the current data set with orthographic stimuli, and whether also the decoder and the network would be fooled by mirror-image letters. The authors also used small case letters as stimuli and therefore have the data. I recommend analyzing this issue with some additional analyses at the single-neuron and population level in order to strengthen this paper’s conclusions even more.

4. The notion of “neuronal recycling” reminded me of the other uniquely human symbol system, the number system. Here, too, humans seem to have inherited a pre-existing brain system (here, the parieto-frontal network) from evolutionary ancestors. This is evidenced by tuned BOLD activity in young children (Kersey & Cantlon, 2017, JN), and neurons responding to numerical quantity in numerically-naïve monkeys (Viswanathan and Nieder, 2013, PNAS) and even crows (Wagener et al., 2018, CurrBiol). Thus, quantitative information is already well encoded in the dorsal visual pathway of innumerate primates, and may bias cultural evolution by determining how quantity can be extracted and linked to visual shapes, aka numerals (see human MTL single-cell recordings by Kutter et al., 2018, Neuron). Perhaps, this would be a suitable discussion point.

Minor comments:
Line 126: What kind of “IT representations” were tested. Please be more specific: single units, multi-units, LFPs,-…? The methods say it is primarily multiunit activity. Please mention this; the reader needs to know this right away.

Line 142. I struggle with the term “decision” to characterize decoder performance. I don’t think decoders “decide” anything, but rather perform based on input data. Wouldn’t “lexical decoder performance” or “classification” be a more appropriate to describe the decoder output?

Line 165. New analysis could be indicated by a line break to form a separate paragraph.

Line 201. New analysis could be indicated by a line break to form a separate paragraph.

Fig. 3: The different grey data points form the different networks are difficult to discriminate and therefore to read. Maybe the authors want to resort to a graded color code.

Line 276: Please briefly indicate how selectivity was tested.

Line 289. Please provide statistical test and p-values for position and laterality.

Line 306. Please provide statistical test and p-value for correlation value comparison.


Reviewer #2 (Remarks to the Author):

Grainger and colleagues (2012, Science) trained baboons to distinguish a set of English words from a set of nonwords, and showed that the baboons developed sensitivity to some types of orthographic structure (e.g., differences in the frequencies of letter combinations). The current work shows that related orthographic distinctions are already reflected in the pre-existing high-level visual (inferotemporal) representations of untrained macaques. This is interesting because it suggests some sort of "proto-representation" of orthographic material, whose homologue in pre-literate children might form the basis for reading acquisition.

I think the reported work is important and should be published, but a particular aspect of how the claims and conclusions are described is highly misleading and must be revised first. In particular, the authors speak of IT representations as supporting "lexical decision"---that is, distinguishing letter strings that are (English) words from those that are not. If true, that would be pretty bizarre as, of course, the animals don't know English. The use of this terminology in the Grainger study was acceptable because the animals were trained on the words; that is, there was a clear experiential basis for distinguishing what letter strings are "words" (trained) versus "nonwords" (novel). This is not the case in the current study.

Here, the untrained animals (and their IT representations) have no way to know whether a particular letter string (FIND) happens to be an English word, whereas another (NART) isn't. Rather, what's going on is that some lower-level orthographic property is *confounded* with what the authors call lexicality. That is, position-specific letter or bigram frequencies happen to be a bit different among the words than among the nonwords---this is unavoidable as the nonwords were scrambled versions of the words---and the animals' IT representations are apparently somewhat sensitive to these orthographic factors (as they impact high-level visual similarity). [The authors really ought to analyze their word and nonword stimuli so as to characterize the relevant orthographic structure more precisely.]

This does *not* mean, as the authors claim, that

Even in untrained non-human primates, the population of IT neurons
contains an explicit (i.e. linearly separable), if still imperfect,
representation of written words. (line 108)

This task requires generalization of a learned lexical classification to
novel orthographic stimuli, rather than the mere memorization of
orthographic properties. (line 148)

These results suggest that lexical decision behavior could be supported by
a sparse, distributed read-out of the IT representation in untrained
monkeys. (line 207)

The population of IT neurons in each subject forms an explicit
representation of orthographic objects. (line 341)

It is the orthographic structure that is linearly separable, not anything like a word representation. That the authors conceive of (and describe) their task as "lexical decision" does not mean that the representations actually support true lexical decision, which is why it is so misleading. The authors need to be much more precise in their description of their methods and results so as to avoid this confusion.

Below are a few more specific comments (indexed by line number):

86: "Here, we reasoned that if orthographic processing abilities are supported by “recycling” primate IT cortex – either by minimal adaptations to the IT representation and/or evolutionary addition of new cortical tissue downstream of IT – then this predicts that the initial state of the IT representation, as measured in naive macaque monkeys, should readily serve as a computational precursor of orthographic processing tests."
* The logic here seems faulty. A region of cortex can be "recycled" to represent stimuli which are *nothing* like what the region normally responds to (and thus could "readily serve as a computational precursor" for). The clear example is the re-routing of visual input to auditory cortex in ferrets (Roe et al., 1992, JNS). It does seem likely that, among regions, the one that becomes response to a new type of stimulus would be the one whose standard responsivity is the most similar, but this need not be very similar - it could just be the best of what are all bad options.

251: "a sparse, distributed read-out of the adult IT representation of untrained non-human primates is sufficient to support many visual discrimination tasks, including ones in the domain of orthographic processing, and that that neural mechanism could be learned with a small number of training examples..."
* The representations themselves presumably require a massive amount of
visual experience to form; what can be learned rapidly is simply the linear
classifier. This should be clarified.

403: "according to constructivist theories of learning (46), experience determines cortical organization, and thus the visual representations that underlie orthographic processing should be largely determined over developmental time-scales by the experience of learning to read. "
* I don't think the constructivist account implies the conclusion here (after "thus"). All that's being shown in the current work is some generalization from past visual experience, which is fully compatible with the constructivist account.

428: "under different variations generative parameters"
* I think this should be "...variations of generative..."

462: "weights in kg"
* Are the actual weights missing here?


Reviewer #3 (Remarks to the Author):

Rajalingham and colleagues investigate the responses in macaque visual cortex (V4 and IT) to visual word stimuli and compare the performance of decoders trained on those data for orthographic processing tasks with performance of Baboons from a prior study. There is much to like about this work – the question is really interesting, the technical aspects of the work are very strong and the manuscript is extremely well written. But there is one fundamental aspect of the work that may limit the interpretability of the findings.

Major Comment

Given that the authors are interested in the question of whether there are potential precursors of orthographic processing in monkeys, it’s surprising that the stimulus conditions were not better matched those typically involved in reading. In the current study the stimuli were presented with a span of 8 degrees of visual angle and letter sizes ranging from 0.8 degrees to 1.6 degrees of visual angle. Yet during typical reading, words may only span less than a degree of visual angle – reading is much more driven by foveal processing than the more extended spatial processing the authors are measuring. Further, it is well known that the span of words and the spacing between letters have strong effects on human behavioral performance in orthographic processing tasks. You could even wonder what human performance would be like on the orthographic processing tasks employed using the same stimulus and temporal presentations rates as in the current study.

These considerations are particularly important given the results – the authors find that the decoders perform better when trained on IT than V4 data. But is this just a reflection of the differences in spatial sensitivity between these areas? With the stimuli presented at these sizes, performance on some of the tasks will require integration over multiple letters which will be better in IT than V4 given the typically decreased spatial sensitivity. Would performance for V4 be better is the stimuli were smaller? To the extent that the results might be affected by the size of the stimuli, the current findings have limited interpretability with relation to orthographic processing that might reflect human reading abilities.

I think it is important for the authors to be clear about why they chose to present the stimuli at the current sizes and what the implications are of this choice for the nature of the results obtained. Further, the details about the stimuli (both size and temporal duration) should be much more prominently stated in the manuscript. These concerns don’t invalidate the results or interpretation, but place significant constraints on them that should be acknowledged and discussed.

Minor Comments

1) Were arrays implanted in both hemispheres of each monkey?
2) More information about the location of the arrays within V4 and IT should be provided (e.g. were the arrays in V4 dorsal or ventral?).
3) Abstract – the notion of a “full rebuild of visual processing” is a bit of an unnecessary straw man. In general, the authors frame the work as if orthographic stimuli came out of nowhere. But orthographic stimuli were designed and developed by humans and the nature of visual processing abilities had to influence the development of orthographies. Some of the text could be tweaked to be a little more nuanced in discussing some of these issues.
4) Line 290 – “substantial tolerance across positions”. It’s not clear what the authors mean here or what evidence they are using. Do they mean selectivity tolerance or just simply effect of stimulus position on average responses? Based on the data provided (e.g. figure 4C), the responses don’t appear to be that tolerant to position to me. Can the authors clarify and be a little more quantitative in their descriptions?

**Author rebuttal, first version:**

**Reviewer #1 (Remarks to the Author):**

The authors recorded neural representations in the primate ventral visual pathway to explore whether such signals would support orthographic classification tasks. Neuronal responses were measured from IT and V4 while each monkey passively viewed rapid streams of images, consisting of alphabet letters, English words, and pseudo-word strings, at the center of gaze. The authors found that IT-based decoders achieved baboon-level performance on these tasks, with a pattern of errors highly correlated to the previously reported primate behavior. This orthographic capacity was also present in the high-layer units of state-of-the-art artificial neural network models of the ventral stream.

This is a great study with fascinating data, and a pleasure to read. The study is a rare and most welcome combination of neuron recordings in monkeys, state-of-the-art decoder analyses and deep network modeling, and comparison with previously published behavioral data from baboons. The results for the first time demonstrate that the nescient state of IT in untrained monkeys has the capacity to support many learned orthographic discriminations characteristic for literate humans. As such, it is a very significant study about the neural precursors of literacy. I only have a few comments I would ask the authors to address prior to publication.

We thank the reviewer for this concise summary and constructive comments, and for his/her interest in this research.

Major comments:

1. Abstract. I think the abstract is badly selling the results. Only two sentences are devoted to the actual findings, while the findings would deserve to be the main part of the abstract. A reader of the abstract would benefit from more information about the actual findings (and less background information).

We thank the reviewer for this suggestion. We have heavily edited the Abstract to match the word-limit of Nature Communications (150 words) while focusing on the results rather than background.

2. Presentation of original data. In the results, the paper right away starts with the presentation of the decoder performance based on neuronal populations. While reading the results, I always wondered about the original neuronal data in the form of histograms, firing rates, or tuning curves. After all, this is what the decoder is working with. Later in Fig2 D,E, the paper speaks about the importance of neuronal selectivity, but again selectivity is presented in a quite derived way. Much later in the results, in the very last chapter, three tuning curves to letters are finally shown (Fig. 4A). While I appreciate this last chapter a lot, I think a reader would benefit from introducing original responses of neurons right at the beginning of the results in order to understand the gist of the derived population measures. I recommend this even if there would be slight redundancy with the last chapter.

We thank the reviewer for this suggestion. To show the raw data, we have added PSTHs from example IT neural sites in Figure 1C. We have edited the text to allude to the complex selectivity of IT sites in the early section of the Results, and reserve the corresponding quantifications for Figure 4. We have additionally included more characterization of individual neural sites in a new Figure 5, which presents new evidence for mirror symmetric responses in IT, as per the reviewer’s suggestion.

3. Test for mirror-image confusion. Humans and animals confuse lateral mirror images, such as the letters “b” and “d”. Previous recordings in macaque IT cortex by Rollhagen & Olson (2000, Science) showed

1

that neurons responded more similarly to members of a lateral mirror-image pair than to members of a vertical mirror-image pair. Similarly, Baylis and Driver (2001, NN) showed that shape-coding cells in the inferotemporal cortex of the monkey generalize between mirror images. It would be fascinating to know whether mirror-image confusion occurred in the current data set with orthographic stimuli, and whether also the decoder and the network would be fooled by mirror-image letters. The authors also used small case letters as stimuli and therefore have the data. I recommend analyzing this issue with some additional analyses at the single-neuron and population level in order to strengthen this paper’s conclusions even more.

We thank the reviewer for this suggestion. Given these very sparse reports, we agree with the reviewer that testing the extent to which “mirror symmetry” is a reliable phenomena across IT of high interest to the field. However, our stimulus set is not ideal for exposing such encoding patterns. Our single-letter image set consisted entirely of uppercase letters (26 upper case letters x 4 positions = 104 images), where no letter is a reflection of another. Similarly, our other image set consisted of strings with both upper and lower case letters (80 strings x 5 variations = 400 images), with no strings with perfect mirror symmetry. Nevertheless, we made our best attempt within our current data to relate the similarity in IT of specific stimulus pairs to their measured (imperfect) pixel-wise symmetry. The results are shown in a new Figure 5 and described at length in the Results section.

To examine this, we then quantified the amount of horizontal and vertical mirror symmetry present in all pairs of letters, using a pixel overlap metric. We trained a multinomial logistic regression decoder on the IT population to identify individual letters, and used this learned decoder to map the IT representation of letter images into a 26 dimensional representation (with one dimension for each of the 26 alphabet letters). We then investigated whether the IT decoder similarity between a given pair of images depended on the amount of horizontal or vertical mirror symmetric overlap between those same images. We observe a modest but statistically significant difference between the horizontal and vertical symmetry dependences, consistent with the previously reported left-right mirror symmetry.

4. The notion of “neuronal recycling” reminded me of the other uniquely human symbol system, the number system. Here, too, humans seem to have inherited a pre-existing brain system (here, the parieto-frontal network) from evolutionary ancestors. This is evidenced by tuned BOLD activity in young children (Kersey & Cantlon, 2017, JN), and neurons responding to numerical quantity in numerically-naïve monkeys (Viswanathan and Nieder, 2013, PNAS) and even crows (Wagener et al., 2018, CurrBiol). Thus, quantitative information is already well encoded in the dorsal visual pathway of innumerate primates, and may bias cultural evolution by determining how quantity can be extracted and linked to visual shapes, aka numerals (see human MTL single-cell recordings by Kutter et al., 2018, Neuron). Perhaps, this would be a suitable discussion point.

We thank the reviewer for this suggestion. We have added a paragraph to the discussion to highlight this similarity. This discussion point also naturally leads to the credible alternatives to this theory, in response to Reviewer 2 and 3’s comments.

Minor comments:

Line 126: What kind of “IT representations” were tested. Please be more specific: single units, multi-units, LFPs,-...? The methods say it is primarily multiunit activity. Please mention this; the reader needs to know this right away.

We have added a sentence in the very beginning of the results section to indicate that these data are largely multiunit: “The majority of the collected neurophysiological data corresponded to multiunit activity, hence we conservatively refer to these neural samples as “neural sites.”

2

Line 142. I struggle with the term “decision” to characterize decoder performance. I don’t think decoders “decide” anything, but rather perform based on input data. Wouldn’t “lexical decoder performance” or “classification” be a more appropriate to describe the decoder output?

We used the term “lexical decision” to describe the task of discriminating between word and pseudo-word stimuli, in a manner consistent with the field. However, as noted by Reviewer 2, this term is inaccurate. We have instead replaced all occurrences of lexical decision by “word classification.” We note that this inaccuracy is not based on the term “decision” but rather the “lexical” component.

Line 165. New analysis could be indicated by a line break to form a separate paragraph. Line 201. New analysis could be indicated by a line break to form a separate paragraph.

We have included line breaks for these and thank the reviewer for pointing these out.

Fig. 3: The different grey data points form the different networks are difficult to discriminate and therefore to read. Maybe the authors want to resort to a graded color code.

We thank the reviewer for this suggestion; we have changed the colors of the CORnet markers.

Line 276: Please briefly indicate how selectivity was tested.

Line 289. Please provide statistical test and p-values for position and laterality.

Line 306. Please provide statistical test and p-value for correlation value comparison.

We have included statistical comparisons to simulated null distributions for letter and position (Figure 4B, C) as well as statistical comparisons across reconstruction correlations (Figure 4D). We have edited the sentence to indicate that selectivity refers to differential response across stimuli.

**Reviewer #2 (Remarks to the Author):**

Grainger and colleagues (2012, Science) trained baboons to distinguish a set of English words from a set of nonwords, and showed that the baboons developed sensitivity to some types of orthographic structure (e.g., differences in the frequencies of letter combinations). The current work shows that related orthographic distinctions are already reflected in the pre-existing high-level visual (inferotemporal) representations of untrained macaques. This is interesting because it suggests some sort of "proto-representation" of orthographic material, whose homologue in pre-literate children might form the basis for reading acquisition.

I think the reported work is important and should be published, but a particular aspect of how the claims and conclusions are described is highly misleading and must be revised first. In particular, the authors speak of IT representations as supporting "lexical decision"---that is, distinguishing letter strings that are (English) words from those that are not. If true, that would be pretty bizarre as, of course, the animals don't know English. The use of this terminology in the Grainger study was acceptable because the animals were trained on the words; that is, there was a clear experiential basis for distinguishing what letter strings are "words" (trained) versus "nonwords" (novel). This is not the case in the current study.

Here, the untrained animals (and their IT representations) have no way to know whether a particular letter string (FIND) happens to be an English word, whereas another (NART) isn't. Rather, what's going on is that some lower-level orthographic property is *confounded* with what the authors call lexicality. That is,

3

position-specific letter or bigram frequencies happen to be a bit different among the words than among the nonwords---this is unavoidable as the nonwords were scrambled versions of the words---and the animals' IT representations are apparently somewhat sensitive to these orthographic factors (as they impact high-level visual similarity). [The authors really ought to analyze their word and nonword stimuli so as to characterize the relevant orthographic structure more precisely.]

This does *not* mean, as the authors claim, that

Even in untrained non-human primates, the population of IT neurons contains an explicit (i.e. linearly separable), if still imperfect, representation of written words. (line 108)

This task requires generalization of a learned lexical classification to novel orthographic stimuli, rather than the mere memorization of orthographic properties. (line 148)

These results suggest that lexical decision behavior could be supported by a sparse, distributed read-out of the IT representation in untrained monkeys. (line 207)

The population of IT neurons in each subject forms an explicit representation of orthographic objects. (line 341)

It is the orthographic structure that is linearly separable, not anything like a word representation. That the authors conceive of (and describe) their task as "lexical decision" does not mean that the representations actually support true lexical decision, which is why it is so misleading. The authors need to be much more precise in their description of their methods and results so as to avoid this confusion.

As noted above in the response to Reviewer 1, we used the term “lexical decision” to describe the task of discriminating between word and pseudo-word stimuli, in a manner consistent with the field (discriminating words from pseudowords). However, we agree with the reviewer that this term is misleading as it constrains the task to be solved by referencing a learned lexicon. In other words, lexical decision is only relevant for recognizing previously learned words.

In the current work, as well as in the behavioral experiments conducted by Grainger and colleagues, the task required that subjects (baboons or decoders) learned to distinguish between words and pseudowords, and generalize to completely novel stimuli. This generalization cannot, by definition, be solved by simply referencing a lexicon, but must instead rely on visual/orthographic features of the novel stimuli. To avoid this confusion, we have replaced the references to “lexical decision” with “word classification” throughout the manuscript. We have additionally included a paragraph in the Discussion to describe the critical differences between a purely lexical behavioral and this invariant visual recognition behavior.

We note that this behavioral domain is analogous to human readers discriminating between words and pseudowords in a language they have not learned (e.g. English-speaking subjects performing the discrimination of French words/pseudowords) or even a script that they have not learned (e.g. English-speaking subjects performing the discrimination of Korean words/pseudowords).

[The authors really ought to analyze their word and nonword stimuli so as to characterize the relevant orthographic structure more precisely.]

For our particular stimulus set, the nature of these distinguishing features has already been explored in quite a lot of detail in existing work. it has been suggested that specific combinations of letters (bigrams)

4

or letters-by-positions may underlie. We have added several sentences in the Discussion to refer to this prior work.

Below are a few more specific comments (indexed by line number):

86: "Here, we reasoned that if orthographic processing abilities are supported by “recycling” primate IT cortex – either by minimal adaptations to the IT representation and/or evolutionary addition of new cortical tissue downstream of IT – then this predicts that the initial state of the IT representation, as measured in naive macaque monkeys, should readily serve as a computational precursor of orthographic processing tests."

* The logic here seems faulty. A region of cortex can be "recycled" to represent stimuli which are *nothing* like what the region normally responds to (and thus could "readily serve as a computational precursor" for). The clear example is the re-routing of visual input to auditory cortex in ferrets (Roe et al., 1992, JNS). It does seem likely that, among regions, the one that becomes response to a new type of stimulus would be the one whose standard responsivity is the most similar, but this need not be very similar - it could just be the best of what are all bad options.

We thank the reviewer for bringing up this important point regarding the definition of “recycling.” We use this term in the manner in which it was originally introduced (Dehaene, 2005; Dehaene & Cohen, 2007). The term “recycling” refers to adaptations of neural mechanisms evolved for older functions to support newer functions, *where the original function is not entirely lost and constrains what the brain can learn*. We note that not all instances of developmental plasticity would meet this definition, as learning may also occur to replace unused functions, as is likely the case in the cited study. We have added this important point to the discussion.

251: "a sparse, distributed read-out of the adult IT representation of untrained non-human primates is sufficient to support many visual discrimination tasks, including ones in the domain of orthographic processing, and that that neural mechanism could be learned with a small number of training examples..."

* The representations themselves presumably require a massive amount of visual experience to form; what can be learned rapidly is simply the linear classifier. This should be clarified.

We thank the reviewer for this clarification – yes, “neural mechanism” referred to here is the “sparse, distributed read-out” of the IT representations (that themselves are likely learned over very large evolutionary and developmental time-scales). We have edited the sentence to clarify this point.

403: "according to constructivist theories of learning (46), experience determines cortical organization, and thus the visual representations that underlie orthographic processing should be largely determined over developmental time-scales by the experience of learning to read. "

* I don't think the constructivist account implies the conclusion here (after "thus"). All that's being shown in the current work is some generalization from past visual experience, which is fully compatible with the constructivist account.

This distinction depends critically on what constitutes as experience, whether only developmental/lifetime or also “experience” over evolutionary time-scales. To avoid this confusion, we have removed the reference to constructivism from this sentence, and replaced it with the explicit alternative that the underlying representations could have been learned largely over developmental timescales.

428: "under different variations generative parameters"

5

* I think this should be "...variations of generative..."

462: "weights in kg"

* Are the actual weights missing here?

We thank the reviewer for catching these typographic errors! We have fixed them in the revision.

**Reviewer #3 (Remarks to the Author):**

Rajalingham and colleagues investigate the responses in macaque visual cortex (V4 and IT) to visual word stimuli and compare the performance of decoders trained on those data for orthographic processing tasks with performance of Baboons from a prior study. There is much to like about this work – the question is really interesting, the technical aspects of the work are very strong and the manuscript is extremely well written. But there is one fundamental aspect of the work that may limit the interpretability of the findings.

Major Comment

Given that the authors are interested in the question of whether there are potential precursors of orthographic processing in monkeys, it’s surprising that the stimulus conditions were not better matched those typically involved in reading. In the current study the stimuli were presented with a span of 8 degrees of visual angle and letter sizes ranging from 0.8 degrees to 1.6 degrees of visual angle. Yet during typical reading, words may only span less than a degree of visual angle – reading is much more driven by foveal processing than the more extended spatial processing the authors are measuring. Further, it is well known that the span of words and the spacing between letters have strong effects on human behavioral performance in orthographic processing tasks. You could even wonder what human performance would be like on the orthographic processing tasks employed using the same stimulus and temporal presentations rates as in the current study.

These considerations are particularly important given the results – the authors find that the decoders perform better when trained on IT than V4 data. But is this just a reflection of the differences in spatial sensitivity between these areas? With the stimuli presented at these sizes, performance on some of the tasks will require integration over multiple letters which will be better in IT than V4 given the typically decreased spatial sensitivity. Would performance for V4 be better is the stimuli were smaller? To the extent that the results might be affected by the size of the stimuli, the current findings have limited interpretability with relation to orthographic processing that might reflect human reading abilities.

I think it is important for the authors to be clear about why they chose to present the stimuli at the current sizes and what the implications are of this choice for the nature of the results obtained. Further, the details about the stimuli (both size and temporal duration) should be much more prominently stated in the manuscript. These concerns don’t invalidate the results or interpretation, but place significant constraints on them that should be acknowledged and discussed.

We thank the reviewer for bringing attention to the factor of stimulus size. We agree that imposing an additional constraint on stimulus size would be an interesting avenue for future research that focuses on putative specialization for reading. The letter sizes that we used (0.8° to 1.6°) are within the range of 0.2° to 2° where human readers exhibit virtually no change in reading abilities. Thus, while small-size stimuli would impose additional constraints that are specific to reading, the stimulus sizes used in our study do not preclude reading. We note that one of the major behavioral targets for this work was the behavioral

6

experiments conducted in baboons by Grainger and colleagues, where the stimuli were presented at larger sizes (~3.4°).

As per the reviewer’s suggestion, we have edited Figure 1, as well as the first paragraph of the Results section, to prominently state the spatial size and temporal duration of the stimuli, and have edited the Methods section to describe the implications.

Minor Comments

1) Were arrays implanted in both hemispheres of each monkey?
2) More information about the location of the arrays within V4 and IT should be provided (e.g. were the arrays in V4 dorsal or ventral?).

We thank the reviewer for this suggestion. We have included a figure panel (Fig 1C) showing the approximate location of arrays in each monkey. Data from each monkey corresponds to one hemisphere, although both hemispheres were sampled across monkeys.

3) Abstract – the notion of a “full rebuild of visual processing” is a bit of an unnecessary straw man. In general, the authors frame the work as if orthographic stimuli came out of nowhere. But orthographic stimuli were designed and developed by humans and the nature of visual processing abilities had to influence the development of orthographies. Some of the text could be tweaked to be a little more nuanced in discussing some of these issues.

We thank the reviewer for highlighting this important point, made in lines 440: “those visual features are already well encoded in the ventral visual pathway of illiterate primates, and may bias cultural evolution by determining which scripts are more easily recognizable and learnable.”

The term “recycling” refers specifically to such adaptations of neural mechanisms evolved for older functions to support newer functions, where the original function is not entirely lost and constrains what the brain can learn. Importantly, not all instances of learning correspond to such recycling, as pointed out by Reviewer #2, who brings attention to occurrences of “recycling”-like mechanisms that are best described as “full rebuilds” (specifically, the emergence of visual activity in ferret auditory cortex).

We have added a paragraph to the Discussion section to highlight this important point.

4) Line 290 – “substantial tolerance across positions”. It’s not clear what the authors mean here or what evidence they are using. Do they mean selectivity tolerance or just simply effect of stimulus position on average responses? Based on the data provided (e.g. figure 4C), the responses don’t appear to be that tolerant to position to me. Can the authors clarify and be a little more quantitative in their descriptions?

We thank the reviewer for bringing up this point. We have added statistical analyses to quantify the extent of tolerance across positions. We observe the IT neurons do not respond perfectly sparsely (i.e. at only one position, sparsity of 100%) nor perfectly uniformly (i.e. at all positions, response sparsity of 0%). We have edited the sentence referring to “substantial tolerance” to be more descriptive.

7

References cited in this response:

Dehaene, S. (2005). Evolution of human cortical circuits for reading and arithmetic: The “neuronal

recycling” hypothesis. In S. Dehaene, J. R. Duhamel, M. Hauser, & G. Rizzolatti (Eds.), *From*

*monkey brain to human brain* (pp. 133–157). Cambridge, Massachusetts: MIT Press. Dehaene, S., & Cohen, L. (2007). Cultural recycling of cortical maps. *Neuron*, *56*(2), 384–398. (17964253).

**Reviewer comments, second version:**

Reviewer #2 (Remarks to the Author):

As original Reviewer 2, my main concern with the initial submission was that, if participants (of any sort) have no basis for knowing that some letter strings are words, it is misleading to characterize any task they perform as distinguishing words from nonwords (i.e., lexical decision). The authors responded to this concern (and one raised by original Reviewer 1) largely by changing the term "lexical decision" to "word classification". While this may allay Reviewer 1's concern about the term "decision", it obviously does nothing to address my concern about "lexical".

Let me express the concern another way. It would be trivial to construct new word and pseudoword stimuli such that all of the current "word classification" results were eliminated. Conversely, it would be trivial to construct two sets of pseudowords such that all of the current "word classification" results held between the sets. So, regardless of what the authors call their task or how they describe their results, what is being classified here is not words versus pseudowords. It is, as I pointed out (and as the authors acknowledge, albeit only in the General Discussion), based on something *confounded* with lexical status. Thus, the paper continues to be misleading.

Now, to be fair, the authors have improved the terminology in a few places, but not nearly enough, as they still say things like

The neural population representation in IT cortex in untrained macaque
monkeys is largely capable, with some supervised instruction, [of
extracting] explicit representations of written letters and words (486).

We here report evidence for a "precursor" of visual word form recognition
in untrained monkeys (493)

Thus, I still think it would be inappropriate to accept the paper for publication. In case the editor disagrees, I've listed below what I think are the changes that are necessary at the very least.

title: Reference to "word form recognition" is misleading. "orthographic representations" would be more accurate.

70: "visual word form recognition" => "orthographic processing"

86: "or sorting out English words from pseudo-words (word classification, also known in human psycholinguistics as the lexical decision task)."
* Please add a following sentence along the lines of "Of course, the animals have no knowledge of English words and so can perform this latter task only on the basis of lower-level orthographic differences among words versus pseudowords."

91: "asking whether adding a simple neural readout machinery on top of the macaque IT representation could produce a neural substrate of orthographic processing"
* Aren't the representations themselves (that are being recorded from) the "neural substrate of orthographic processing"? The read-out is just a way to show that those representations contain particular types of information. (Using a "neural-like" read-out mechanism is useful because it ensures that the information is plausibly accessible to other neural representations.)

99: " the population of IT neurons contains an explicit (i.e. linearly separable), if still imperfect, representation of written words"
* "...representation of orthographic stimuli"

188: "the distributed neural representation in macaque IT cortex is sufficient to explain the word classification behavior of baboons, which itself was previously found to be correlated with human behavior (17)".
* Please add "...correlated with human behavior with the same stimuli" as, of course, baboon behavior is unlikely to be correlated with human behavior in lexical decision in general.

273: "printed words" => "printed letter strings"

320: "this was difference was" => "this difference was"

342: "a small proportion of neurons in IT exhibit “mirror-symmetric” tuning....this phenomenon has been hypothesized to underlie the left-right inversion errors of children learning to read.... 361: "pixel-level reflectivity"
* Mirror confusions in letter perception in children are not about the pixel-level* similarity of the alternatives (e.g, b vs. d), as then letters like "c" and "o" would be nearly equally often confused, which they're not. The relevant similarity is at a higher, structural level. Accordingly, I don't think the analysis in this section provides any information about the basis of the relevant phenomena in children.

402: "we hypothesized that the neural representations that directly underlie human orthographic processing abilities must be strongly constrained..."
* Maybe "are" rather than "must be"

404: "Here, we observed that orthographic information was explicitly encoded in sampled populations of spatially distributed IT neurons in naïve, illiterate, non-human primates. Our results are consistent with the hypothesis that the population of IT neurons in each subject forms an explicit representation of orthographic objects"
* I don't know what the authors mean by "explicit" here, but just being decodable by a linear classifier at an above-chance rate surely isn't sufficient. Both uses should be dropped.

486: "is largely capable...to extract"
* Either "able...to extract" or "capable...of extracting"

486: "extract explicit representations of written letters and words"
* same point about "explicit"; should be something like "representing orthographic information"

493: "we here report evidence for a “precursor” of visual word form recognition in untrained monkeys"
* no you don't; how about "precursor of orthographic representations in untrained monkeys"

493: "This finding fits with nativist views of cognitive development"
* What? The "precursor" representations could themselves be learned from earlier general visual experience (Changizi et al., 2006, ref 24). There is nothing specifically nativist here.


Reviewer #3 (Remarks to the Author):

The authors have failed to address my concerns meaningfully. The main reason I raised the question of stimulus size is to ask whether the differences the authors observed between V4 and IT could reflect the specific choice of stimulus size – would the results in V4 have looked different with smaller stimuli that required less integration over space? I think this is an important question with regard to the interpretation of the results. I refer them back to my original comments.

To justify the relatively large stimuli the authors point to the baboon work, but if they wanted to match the baboon work why not use the same size stimuli? If the point is to provide insight into human reading then why not use stimuli that match the typical text size (closer to 0.3 degrees for letter width)? Can the authors please explain their choice – if it was somewhat arbitrary then they should just be transparent, rather than try and justify post-hoc.

The authors reference work suggesting that human readers show no variation in reading for stimuli with letter sizes in the range of 0.2 to 2 degrees. But it’s not completely clear how relevant this work is for the current stimuli, given that the current stimuli were upper case letters, both words and non-words, and spacing between letters presumably varied with letter size since it appears that string width was kept constant.

The authors state that they have addressed the implications of stimulus size in the Methods, but I found no relevant discussion there, and I think this is an issue that really belongs in the Discussion.

**Author rebuttal, second version:**

**Reviewer #2 (Remarks to the Author):**

As original Reviewer 2, my main concern with the initial submission was that, if participants (of any sort) have no basis for knowing that some letter strings are words, it is misleading to characterize any task they perform as distinguishing words from nonwords (i.e., lexical decision). The authors responded to this concern (and one raised by original Reviewer 1) largely by changing the term "lexical decision" to "word classification". While this may allay Reviewer 1's concern about the term "decision", it obviously does nothing to address my concern about "lexical".

Let me express the concern another way. It would be trivial to construct new word and pseudoword stimuli such that all of the current "word classification" results were eliminated. Conversely, it would be trivial to construct two sets of pseudowords such that all of the current "word classification" results held between the sets. So, regardless of what the authors call their task or how they describe their results, what is being classified here is not words versus pseudowords. It is, as I pointed out (and as the authors acknowledge, albeit only in the General Discussion), based on something *confounded* with lexical status. Thus, the paper continues to be misleading.

Now, to be fair, the authors have improved the terminology in a few places, but not nearly enough, as they still say things like

“The neural population representation in IT cortex in untrained macaque monkeys is largely capable, with some

supervised instruction, [of extracting] explicit representations of written letters and words (486).”

“We here report evidence for a "precursor" of visual word form recognition in untrained monkeys (493)”

Thus, I still think it would be inappropriate to accept the paper for publication. In case the editor disagrees, I've listed below what I think are the changes that are necessary at the very least.

We thank the reviewer for clarifying this comment, and for the suggested changes in the manuscript text. We believe that we have now done all the requested changes. Details of each change are provided below.

We believe these suggested changes are consistent with the findings we present, namely that the representation in IT is sufficient to support a battery of orthographic processing tasks, including the discrimination between two operationally defined set of orthographic stimuli (stimuli sampled from the set of four letter English words, and stimuli sampled from the set of four letter pseudo-words).

title: Reference to "word form recognition" is misleading. "orthographic representations" would be more accurate.

We have changed the title to “**A potential cortical precursor of orthographic processing in untrained monkeys.”**

70: "visual word form recognition" => "orthographic processing"
We have made this suggested edit.

86: "or sorting out English words from pseudo-words (word classification, also known in human psycholinguistics as the lexical decision task)."

* Please add a following sentence along the lines of "Of course, the animals have no knowledge of English words and so can perform this latter task only on the basis of lower-level orthographic differences among words versus pseudowords."

We thank the reviewer for this suggestion. We have added the following sentence: “Given that animals have no semantic knowledge of English words, ‘word classification’ here refers to a visual (rather than lexical) discrimination task, i.e. the ability to categorize letter strings as “words” or “pseudo-words” on the basis of visual and/or orthographic features, generalizing to novel letter strings drawn from the same generative distributions.”

99: " the population of IT neurons contains an explicit (i.e. linearly separable), if still imperfect,

representation of written words"

* "...representation of orthographic stimuli"

We have made this suggested edit.

188: "the distributed neural representation in macaque IT cortex is sufficient to explain the word classification behavior of baboons, which itself was previously found to be correlated with human behavior (17)".

* Please add "...correlated with human behavior with the same stimuli" as, of course, baboon behavior is unlikely to be correlated with human behavior in lexical decision in general.

We have added this clarification, and thank the reviewer for this suggestion.

273: "printed words" => "printed letter strings"
We have made this suggested edit.

493: "we here report evidence for a “precursor” of visual word form recognition in untrained monkeys"

* no you don't; how about "precursor of orthographic representations in untrained monkeys"

We have made this suggested edit.

We understand the reviewer’s claim, that words are defined by their existence in a lexicon, rather than their visual features. However, this does not negate the statistical regularities that exist in the visual features of words in our (English) lexicon. Moreover, the fact that one could potentially construct arbitrary new word stimuli that do not exhibit those regularities, does not negate our claim as our target of investigation is the distribution of existing words constructed and constrained over the course of civilization. For the existing distribution of four-letter English words, the orthographic/visual regularities are sufficient such that animals and decoders can accurately, albeit not perfectly, discriminate between words and pseudo-words, even if those specific words have not been seen before. In this work, we show that this behavior (termed “word classification” and distinct from “lexical decision”) can be supported by simple linear read-outs from the IT cortex of illiterate monkeys.

Analogously, humans, animals and machines can learn to visually discriminate between other object categories, such as faces, camels, spoons, fractals, “greebles” and “batons.” Investigating how such visual discriminations are supported does not force one to test whether humans/animals/machines understand other, unrelated semantic relationships pertaining to those object categories. In other words, a monkey or machine may not “understand” spoons (an unclear, ill-posed behavioral criterion) but can still visually discriminate spoons from forks, based on the visual features discriminative of these two categories. This behavior is correctly described as “spoon versus fork discrimination.” While it is unclear why “words” and “pseudowords” should not follow this simple logic, we understand that many readers may be similarly confused regarding visual versus lexical discriminations. Thus, we hope that the changes described above more accurately describe our results as pertaining to visual discriminations rather than lexical, semantic, or other behaviors.

Below, we address the reviewer’s other points, unrelated to the use of the term “word.”

91: "asking whether adding a simple neural readout machinery on top of the macaque IT representation could produce a neural substrate of orthographic processing"

* Aren't the representations themselves (that are being recorded from) the "neural substrate of orthographic processing"? The read-out is just a way to show that those representations contain particular types of information. (Using a "neural-like" read-out mechanism is useful because it ensures that the information is plausibly accessible to other neural representations.)

As stated on line 126, linear decoders are not simply an analysis tool for extracting information from neural populations, but as simple, biologically plausible approximation of downstream neuronal mechanisms that link neural activity to behavior. Thus, the neural substrate of orthographic processing refers to visual representations + decoders.

320: "this was difference was" => "this difference was"

402: "we hypothesized that the neural representations that directly underlie human orthographic

processing abilities must be strongly constrained..."

* Maybe "are" rather than "must be"

486: "is largely capable...to extract"

* Either "able...to extract" or "capable...of extracting"

We thank the reviewer for catching these typographical errors, which we have edited appropriately.

342: "a small proportion of neurons in IT exhibit “mirror-symmetric” tuning....this phenomenon has been hypothesized to underlie the left-right inversion errors of children learning to read.... 361: "pixel-level reflectivity"

* Mirror confusions in letter perception in children are not about the pixel-level* similarity of the alternatives (e.g, b vs. d), as then letters like "c" and "o" would be nearly equally often confused, which they're not. The relevant similarity is at a higher, structural level. Accordingly, I don't think the analysis in this section provides any information about the basis of the relevant phenomena in children.

We incorporated this analysis based on the suggestions of Reviewer #1, and we remain careful to not make any strong inferences from the current data: “These results provide modest evidence that a read­out of the IT representation of letters is consistent with previously reported left-right mirror symmetry, and suggest that a targeted experiment designed to directly test this hypothesis is warranted.”

That being said, our analyses attempt to quantify mirror symmetry using delta-R, a difference of pixel similarity after horizontal versus vertical reflections, rather than a simple pixel overlap. As can be seen in Figure 5, pairs of letters with strong pixel overlap (such as “C” and “Q”) or strong vertical symmetry (“A” and “V”) do not have large delta-R. We have edited the text to make this clear.

404: "Here, we observed that orthographic information was explicitly encoded in sampled populations of spatially distributed IT neurons in naïve, illiterate, non-human primates. Our results are consistent with the hypothesis that the population of IT neurons in each subject forms an explicit representation of orthographic objects"

* I don't know what the authors mean by "explicit" here, but just being decodable by a linear classifier at an above-chance rate surely isn't sufficient. Both uses should be dropped.

486: "extract explicit representations of written letters and words"

* same point about "explicit"; should be something like "representing orthographic information"

As stated on line 109, the definition that we and others use for the word “explicit” both here and across prior work is indeed based on linear separability (e.g. DiCarlo et al., 2012; Hong et al, 2016; Kell et al., 2018;). We have edited the text to make this definition clearer.

493: "This finding fits with nativist views of cognitive development"

* What? The "precursor" representations could themselves be learned from earlier general visual

experience (Changizi et al., 2006, ref 24). There is nothing specifically nativist here.

As in the last round of reviews, this confusion depends critically on what constitutes “learning”, whether only developmental/lifetime or also “learning” over evolutionary time-scales. To avoid this confusion, we have removed this reference to “nativism” and have replaced it with: “This finding is consistent with the hypothesis that learning rests on pre-existing neural representations which it only partially reshapes.”

**Reviewer #3 (Remarks to the Author):**

The authors have failed to address my concerns meaningfully. The main reason I raised the question of stimulus size is to ask whether the differences the authors observed between V4 and IT could reflect the specific choice of stimulus size – would the results in V4 have looked different with smaller stimuli that required less integration over space? I think this is an important question with regard to the interpretation of the results. I refer them back to my original comments.

Apologies – we now see that we did not correctly interpret the reviewer’s previous comments regarding this issue, and we thank the reviewer for clarifying this point.

The reviewer is concerned that the visual stimuli are optimized in size for a read-out from IT better than V4, but that this effect would change as a function of stimulus size. For example, he/she is concerned that for smaller stimuli, linear decodes from earlier visual cortical areas (e.g. V4) would perform better than those from IT. This is an important control that, while inconsistent with prior work in the domain of object recognition, should be considered. To test this, we have added new analyses, a new figure panel and corresponding text changes investigating the role of stimulus size on decoding performance in V4 and IT.

We compared the performance of V4 and IT populations over the battery of orthographic processing tests (including letter recognition, bigram detection, and word classification), as a function of letter size. Figure shows the scatter over all tests, and the average difference in performance between IT and V4 for each letter size (0.8, 1.2, and 1.6 deg). We observe that linear decodes from IT consistency outperform linear decodes from V4 over these tested letter sizes, which span the range of letter size relevant for “normal” human behavior.

To justify the relatively large stimuli the authors point to the baboon work, but if they wanted to match the baboon work why not use the same size stimuli? If the point is to provide insight into human reading then why not use stimuli that match the typical text size (closer to 0.3 degrees for letter width)? Can the authors please explain their choice – if it was somewhat arbitrary then they should just be transparent, rather than try and justify post-hoc.

Prior work suggests that the ventral stream is largely focused on the central 10° of the visual field, and our typical neurophysiological experiments consist of images presented to span the central 8° (as stated on line 525). Consistent with this prior work, we adapted the stimuli used for the baboon work in Grainger et al. (~14° for the letter string) to images that span the central 8°.

As the reviewer may be aware of, it is substantially more difficult to measure reliable neurophysiological signals with precisions of 0.1deg in awake and behaving monkeys (or humans), given the variability in gaze fixation, fixational eye movements, and in gaze tracking. Such precision is only feasible in an anesthetized preparation.

We have edited the Methods section to include this information.

The authors reference work suggesting that human readers show no variation in reading for stimuli with letter sizes in the range of 0.2 to 2 degrees. But it’s not completely clear how relevant this work is for the current stimuli, given that the current stimuli were upper case letters, both words and non-words, and spacing between letters presumably varied with letter size since it appears that string width was kept constant.

As shown in Figure 1, we did not keep the string width constant; the spacing between letters was proportional to the letter sizes themselves. We have added this information the Methods to clarify this point.

We include this reference simply to state that our stimulus size is within the “normal” range where human readers exhibit no behavioral difference. If the stimuli had been presented at much smaller (e.g. 0.05 deg) or much bigger (e.g. 10 deg) sizes, we hope the reviewer would agree that the results may be less relevant for investigating potential precursors of the orthographic stage of human reading.

The authors state that they have addressed the implications of stimulus size in the Methods, but I found no relevant discussion there, and I think this is an issue that really belongs in the Discussion.

Thank you for encouraging us to include this. As discussed above, and thanks to the reviewer’s suggestion, we have added a new figure panel to discuss the implications of stimulus size (see above for details).

**Reviewer comments, third version:**

Reviewer #3 (Remarks to the Author):

The authors have now fully addressed my concerns and I appreciate their additional analyses and discussion.

**Author rebuttal, third version:**

**Reviewer #3 (Remarks to the Author):**

*The authors have now fully addressed my concerns and I appreciate their additional analyses and discussion.*

We’re happy that the reviewer’s concerns are now addressed, and we once again thank all reviewers for their valuable and constructive feedback.
